# The Role of Packing, Dispersion, Electrostatics, and Solvation in High‐Affinity Complexes of Cucurbit[*n*]urils with Uncharged Polar Guests

**DOI:** 10.1002/chem.202200529

**Published:** 2022-05-25

**Authors:** Laura M. Grimm, Sebastian Spicher, Boryslav Tkachenko, Peter R. Schreiner, Stefan Grimme, Frank Biedermann

**Affiliations:** ^1^ Institute of Nanotechnology Karlsruhe Institute of Technology Hermann-von-Helmholtz Platz 1 76344 Eggenstein-Leopoldshafen Germany; ^2^ Mulliken Center for Theoretical Chemistry Institute of Physical and Theoretical Chemistry University of Bonn Beringstraße 4 53115 Bonn Germany; ^3^ Institute of Organic Chemistry Justus Liebig University Heinrich-Buff-Ring 17 35392 Giessen Germany

**Keywords:** cucurbiturils, density functional calculations, diamondoids, host-guest systems, isothermal titration calorimetry, thermodynamics

## Abstract

The rationalization of non‐covalent binding trends is both of fundamental interest and provides new design concepts for biomimetic molecular systems. Cucurbit[*n*]urils (CB*n*) are known for a long time as the strongest synthetic binders for a wide range of (bio)organic compounds in water. However, their host‐guest binding mechanism remains ambiguous despite their symmetric and simple macrocyclic structure and the wealth of literature reports. We herein report experimental thermodynamic binding parameters (Δ*G*, Δ*H*, TΔ*S*) for CB7 and CB8 with a set of hydroxylated adamantanes, di‐, and triamantanes as uncharged, rigid, and spherical/ellipsoidal guests. Binding geometries and binding energy decomposition were obtained from high‐level theory computations. This study reveals that neither London dispersion interactions, nor electronic energies or entropic factors are decisive, selectivity‐controlling factors for CB*n* complexes. In contrast, peculiar host‐related solvation effects were identified as the major factor for rationalizing the unique behavior and record‐affinity characteristics of cucurbit[*n*]urils.

## Graphical abstract

**Experimentally obtained (ITC)** and computed (DFT) thermodynamic binding parameters (Δ*G*, Δ*H*, *T*Δ*S*) are reported for CB7‐ and CB8‐complexes with uncharged, rigid, and spherically‐shaped guests. The simplicity and diversity of the model system enabled a deep analysis of the energetic driving forces and uncovered the importance of solvation energies. Electrostatic interactions and London dispersion forces are not the selectivity controlling factors.

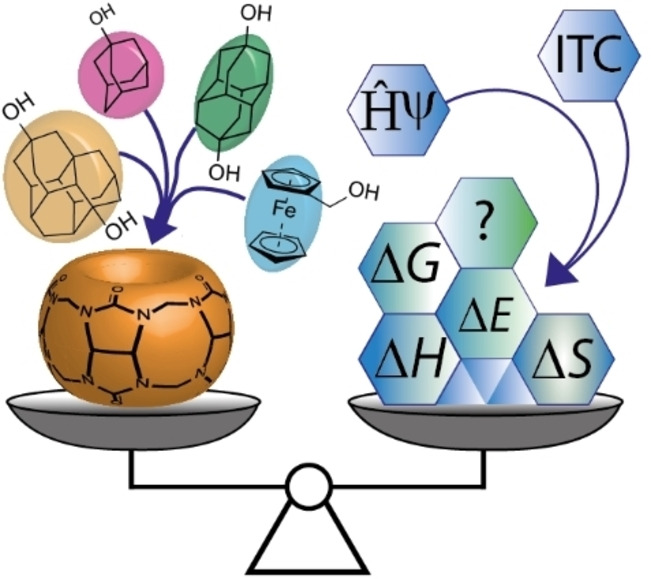

## Introduction

Cucurbit[*n*]urils (CB*n*, *n*=5–8, 10, 14) are glycoluril‐based barrel‐shaped synthetic hosts^[1]^ that are known as record‐holding high‐affinity binders for a wide range of substance classes, for example, amino acids and derivatives, peptides, steroids, drugs, hydrocarbons, and even certain proteins in water.^[2]^ Extremely high affinities have been reported for CB7 with some positively charged adamantane and ferrocene derivatives (∼10^14^ M^−1^) reaching astonishing values of up to 10^17^ M^−1^ for amino‐substituted diamantanes as guests.^[3]^ These findings are even more surprising when acknowledging that CB*n*‐type hosts were discovered by chance with CB6 being first synthesized already in 1905.^[4]^ Still, this host class outperforms most other artificial receptors and synthetic hosts in terms of binding strength in aqueous media. Thus, understanding the origin of this remarkable binding strength of CB*n* is of great interest as it promises unraveling of transferable design principles that can be applied to the design of other synthetic binders for use in materials chemistry,^[5]^ drug delivery,^[6]^ or molecular sensing applications.[^2b^, ^7^] Fortunately, CB*n* are biocompatible and non‐toxic.^[8]^

While the particularly strong binding of positively charged guests to cucurbit[*n*]urils has been tentatively attributed to stabilizing ion‐dipole interactions between the carbonyl‐fringed CB*n* portals and the cation charge center,[^1^, ^9^] this effect cannot explain that also weakly dipolar and charge‐neutral guests such as steroids are strongly complexed (*K*_a_>10^6^ M^−1^)^[10]^ by this promiscuously binding macrocyclic family. Furthermore, it should be noted that CB*n* complex formations are generally highly enthalpically favored (up to −22 kcal mol^−1^) which does not differ significantly between charged and non‐charged guests.[^3^, ^11^] Special features of the guests, for example, hydrophobic solvation effects of convex solutes,^[12]^ can only partly rationalize these trends as, for instance, other synthetic receptors with concave binding cavities such as cyclodextrins or cavitands are weaker binders for the same types of guests.^[13]^ Moreover, it is intriguing that the highest binding affinities of CB*n* complexes known this far follow the trend CB6 < CB7 > CB8.

Different explanations have been given for rationalizing the peculiar binding properties of CB*n*. For CB*n*‐complex formation with uncharged, apolar guests in aqueous media, London dispersion interactions^[14]^ or strong hydrophobic contributions (high‐energy cavity water release^[15]^ or differential cavitation effects^[14b]^) come to mind. Additional factors such as the binding entropy may also play an important role but are difficult to estimate both experimentally and theoretically.^[16]^ For instance, hydrocarbon or peptidic guests for CB7 and CB8 that were studied in depth, for example, by Nau, Isaacs, Urbach, Kim, and others,^[17]^ can adopt folded conformations both in solution and in the cavity of CB*n*, which may contribute a sizeable entropic factor to the experimentally determined binding free energy.

Access to experimental binding free enthalpies (Δ*H*) would be of additional value for dissecting the driving forces for binding. However, such data are typically only available for CB*n*‐complexes with polar or charged and thus water‐soluble guests (see, for instance, the extensive reports by Urbach, Nau, Scherman, and us).[^3^, ^11c^, ^15b^, ^18^] For such compounds accounting for the energetic contributions of guest desolvation can be difficult.^[19]^ Finally, it should also be noted that both commercial and self‐made CB*n* samples inevitably contain sizeable amounts of residual salts unless particular protocols have been followed – one is employed herein – to desalinate the system. Due to the rather high affinity of CB*n* for metal cations (*e. g*., log *K*_a_(Na^+^ ⋅ CB7)=3.41),^[20]^ salts within CB*n* samples (several equivalents of NaCl per CB*n* equivalent are not uncommon) may thus have influenced the reported thermodynamic host‐guest binding parameters of CB*n*‐guest complexes depending on the experimental conditions employed.

**Figure 1:**
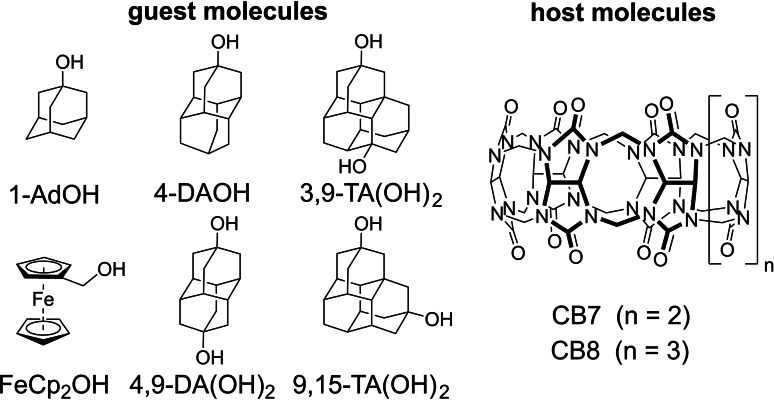
Chemical structures of the water‐soluble guest and host molecules investigated in this study.

In order to shed more light onto the driving forces for CB*n*‐guest complexation and to arrive at generalizable receptor design principles, we devised and report herein a systematic and accurate experimental and computational study of the binding behavior for some of the simplest conceivable model guests – nearly spherical or ellipsoidal, shape‐rigid, and charge‐neutral organic compounds – with the hosts CB7 and CB8 in deionized water (Figure 1).

## Results

### Experimental design

In order to address the aforementioned limitations of other previously reported systematic binding studies with cucurbit[*n*]urils, and to provide a different angle for the interpretation of experimental versus computed binding trends, we aimed towards the analysis of the possible most simple yet still structurally diverse set of CB*n*‐guest complexes. Moreover, we restricted our attention to systems that can be characterized by isothermal titration calorimetry (ITC) experiments in order to access the binding free energy (Δ*G*), the binding enthalpy (Δ*H*), and the entropic contribution (TΔ*S*) of the binding event.

Based on the fact that CB*n* possess a nearly spherical and symmetric cavity (the point groups of non‐deformed CB7 and CB8 are *D*_7h_ and *D*_8h_, respectively), we reasoned that highly symmetric, nearly spherical or ellipsoidal shaped guests that are conformationally locked are the most suited to mitigate entropy‐impacting conformational, rotational, and vibrational effects upon host‐guest complexation. While perfectly spherical noble gases have been reported as the ideal model guests for CB5,^[14b]^ there are no mono‐ or diatomic species conceivable as efficiently CB7‐ or CB8‐binding guests due the otherwise resulting large size mismatches.

Adamantane, diamantane, and triamantane as well as ferrocene derivatives appeared to provide the best possible compromise between rigidity, symmetry, and structural simplicity. Adamantane and higher diamondoids^[21]^ have been instructive in explaining and understanding London dispersion (LD) effects because they are highly rigid and almost perfectly isotropically polarizable.^[22]^ They can readily be functionalized; their alcohol derivatives as used here are reasonably soluble in a large variety of solvents, including water.^[23]^ Thus, we have selected the hydroxyl‐derivatives of adamantane, di‐, and triamantane in order to ensure sufficient aqueous solubility (≥100 μM needed for ITC experiments) of the guests in deionized water while minimizing the influences of complicating factors such as guest charges on the binding energy decomposition.

### Preparation of the compounds and aqueous solutions

The diamantane (4‐DAOH and 4,9‐DA(OH)_2_) and triamantane (3,9‐TA(OH)_2_ and 9,15‐TA(OH)_2_) alcohols were synthesized according to earlier reports.^[23]^

While complexes of CB7 and CB8 with 1‐adamantanol (1‐AdOH) and ferrocenylmethanol (FeCp_2_OH) have already been reported in literature,[^3^, ^10^, ^11c^] we also reevaluated the binding parameters of these complexes because CB*n* samples often contain various impurities, such as water, hydrogen chloride, and ammonium and alkali metal ion salts, typically introduced in the course of their preparation and purification, which can significantly influence the binding parameters.^[24]^ Herein, CB*n* samples were desalinated by careful dialysis prior to use. The concentrations of such CB*n* solutions were then accurately determined by an optical spectroscopy‐based titration with MDAP (for CB7) and MPCP (for CB8) as indicator dyes, following previous literature reports.^[25]^

We have repeatedly observed that stock solutions of such desalted CB7 and CB8 samples in deionized water appeared to partially decompose after several weeks standing at room temperatures indicated by visible flocculation and concentration reduction whereas stock solutions of non‐desalted CB7/CB8 samples are known to be long‐term stable which was also confirmed in our laboratory. Thus, desalted CB*n*‐stock solutions used for fundamental binding studies in deionized water were prepared freshly every couple of days. In addition, calorimetric binding studies (see below) were repeated at different times to further ensure the reproducibility of the results.

### ^1^H NMR experiments

Firstly, ^1^H NMR experiments were carried out to assess if inclusion complexes formed with the candidate high‐affinity guests. The CB*n* cavities constitute an NMR‐shielding region whereas guest residues located outside of the carbonyl‐fringed rims of CB*n* generally experience deshielding.[^9^, ^17a^, ^26^] As expected, an upfield shift and a signal broadening of the adamantane and diamantane protons were observed for both the CB7 and CB8 complexes (see Supporting Information, Figures S1–S4). The NMR data suggest that generally inclusion complexes formed. Moreover, ^1^H NMR experiments verified that a 1 : 1 complexation stoichiometry is adopted for all confirmed inclusion complexes. Specifically, the larger upfield shifts of the methine protons than of the methylene protons of 4,9‐DA(OH)_2_ suggest that the methine protons are buried more deeply in the CB*n* cavity than the methylene protons, as one would expect for a symmetric inclusion complex. Only the interactions of the spacious guests 3,9‐TA(OH)_2_ and 9,15‐TA(OH)_2_ with CB7 were an exception where the experimental evidence ruled out the positioning of the guest in the hosts’ cavity. Unfortunately, the solubility of 9,15‐TA(OH)_2_ in pure water was too low for ITC experiments; this guest candidate was thus excluded from the binding study even though its complexation with CB8 has been confirmed by ^1^H NMR (see Supporting Information, Figure S3).

### ITC experiments

Isothermal titration calorimetry (ITC) experiments were carried out to characterize thermodynamic parameters such as affinity, enthalpy, and entropy, for the combinations of CB7 or CB8 with the inclusion‐complex forming neutral guests.

**Figure 2:**
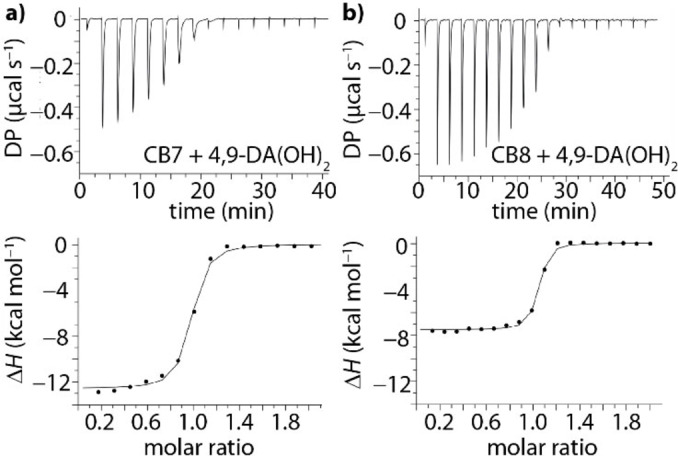
ITC isotherms (dilution heat corrected) for the titration of 4,9‐DA(OH)_2_ (*c*=0–90 μM/*c*=0–45 μM) to a) CB7 (*c*=43 μM) and b) CB8 (*c*=20 μM) at 25 °C.

In a typical experiment, aqueous solutions of desalted CB7 or desalted CB8 were loaded into the cell and titrated with approx. 10× higher concentrated guest solutions that were prepared from the same deionized water source. Figure 2 displays a representative ITC graph for the complex formation of 4,9‐DA(OH)_2_ with CB7 and CB8 (see also Supporting Information, Figures S5–S9).

The binding enthalpy (Δ*H*_exp_) was accurately available (typical StDev≤0.4 kcal mol^−1^, Supporting Information, Figure S5) from direct titration experiments in all cases owing to the sigmoidal or step‐like curve shape of the ITC plots. Most binding constants (and thus binding free energies, Δ*G*_exp_) were accessible by direct curve fitting of the ITC traces but the affinities of the ultrahigh‐affinity complexes of CB7 ⋅ FeCp_2_OH and CB7 ⋅ 1‐AdOH had to be determined by competitive methods (see Supporting Information, Figures S9 and S10) Finally, entropic contributions to binding (−TΔ*S*_exp_) were obtained from the directly measured Δ*H*_exp_ and Δ*G*_exp_ values through −*T*Δ*S=*Δ*G*−Δ*H*. In contrast to other indirect methods that estimate enthalpic and entropic binding contributions from the temperature dependence of the binding affinity (van't Hoff method^[27]^), which are often practically unsuitable for supramolecular systems due to large errors, the herein pursued calorimetric approach is expected to provide accurate values (within a≤0.8 kcal mol^−1^ error) for Δ*H*_exp_ and −*T*Δ*S*_exp_, allowing for a meaningful comparison and scrutinization of possible binding models. Repetition experiments were carried out including those with newly prepared stock solutions in order to estimate the experimental error values (see also Supporting Information, Figure S5). The so obtained thermodynamic parameters, i. e., log*K*_a_, Δ*G*_exp_, Δ*H*_exp_, and −*T*Δ*S*_exp_, are tabulated in Table 1.

**Table 1:** Summary of the binding parameters for the complexation of un‐charged guests with desalined CB7 or desalined CB8 in deionized water, 25 °C, determined by ITC. The data was averaged from at least three dilution heat corrected experiments. Typical errors are 0.2 in log *K*_a_, 0.4 kcal mol^−1^ in Δ*H*_exp_ and Δ*G*_exp_, and 0.8 kcal mol^−1^ in −TΔ*S*_exp_.

host ⋅ guest complex	PC^[a]^ [%]	log *K*_a_^[b]^	Δ*G*_exp_^[c]^ [kcal mol^−1^]	Δ*H*_exp_^[d]^ [kcal mol^−1^]	−*T*Δ*S*_exp_^[e]^ [kcal mol^‐1^]
CB7 ⋅ 1‐AdOH^[f]^	63	10.4	−14.2	−19.0	4.8
CB7 ⋅ 4‐DAOH	79	6.8	−9.3	−12.0	2.8
CB7 ⋅ 4,9‐DA(OH)_2_	79	7.1	−9.6	−12.6	3.0
CB7 ⋅ FeCp_2_OH^[g]^	64	9.4	−12.8	−21.1	8.3
CB8 ⋅ 1‐AdOH	42	6.8	−9.3	−8.1	−1.2
CB8 ⋅ 4‐DAOH	54	6.6	−9.1	−7.8	−1.2
CB8 ⋅ 4,9‐DA(OH)_2_	57	7.2	−9.9	−7.7	−2.3
CB8 ⋅ 3,9‐TA(OH)_2_	60	7.0	−9.5	−12.7	3.2
CB8 ⋅ FeCp_2_OH	42	6.6	−9.0	−13.1	4.2

[a] Packing coefficient. [b] Logarithmic binding affinity. [c] The experimental Gibbs free binding energy was obtained via Δ*G*_exp_=−*RT*ln*K*_a_. [d] Experimental binding enthalpy. [e] Experimental entropic contributions to complex formation obtained via −TΔ*S*_exp_=Δ*G*_exp_−Δ*H*_exp_. [f] Binding affinity was determined by fluorescence‐based titration, see Supporting Information. [g] Binding affinity was determined by multistep ITC with phenylalanine as competitor. Error values are expected to be not larger than 0.4 in log*K*_a_, 0.8 kcal mol^−1^ in Δ*H*_exp_ and Δ*G*_exp_, and 1.6 kcal mol^−1^ in −*T*Δ*S*_exp_.

**Figure 3:**
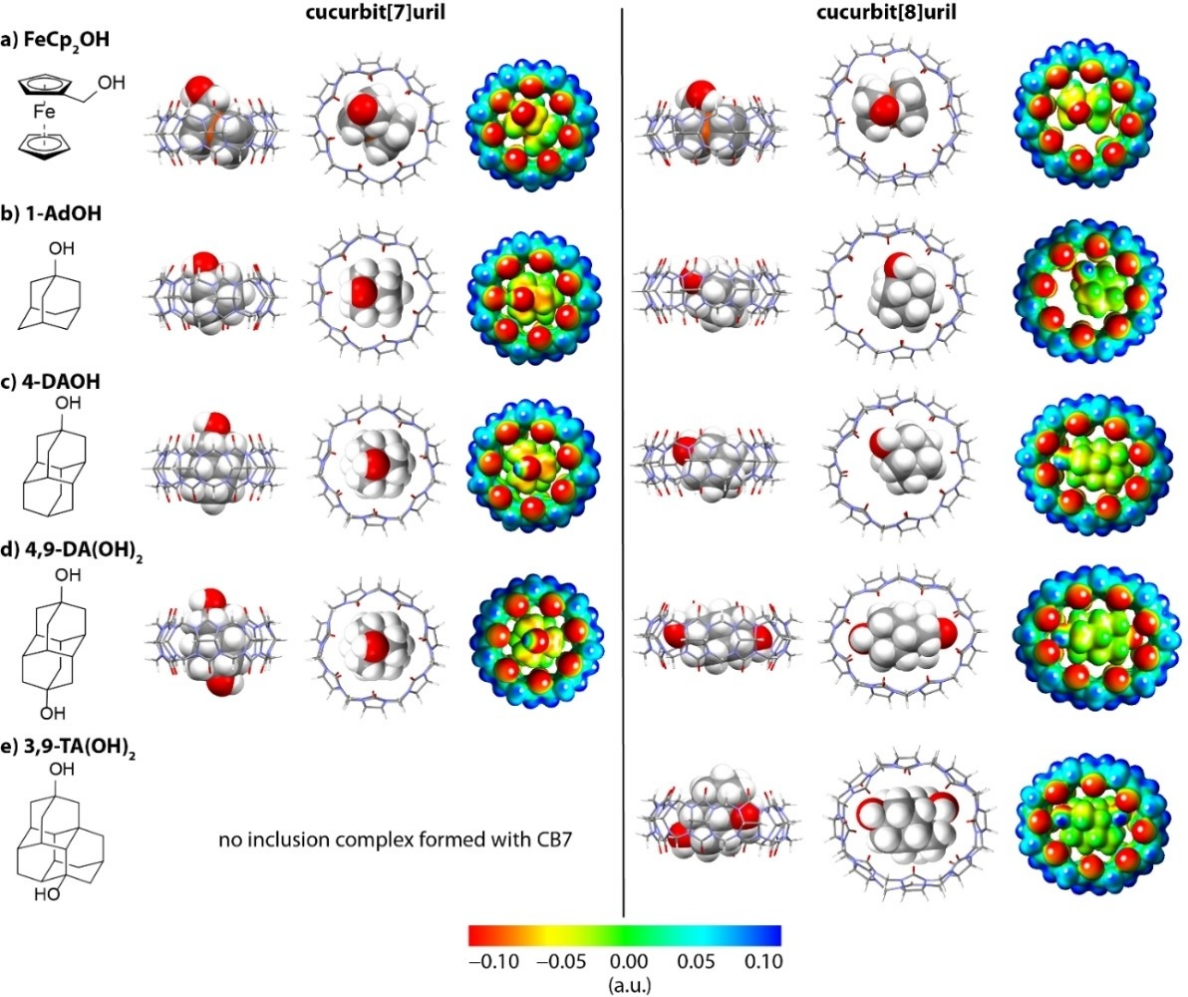
Quantum mechanically calculated (r^2^SCAN‐3c/DCOSMO‐RS) structures for the within this work investigated complexes and their corresponding electrostatic potential maps. The structures shown have the largest contribution to the calculated binding free energy. The following complexes were found to contain one or two hydrogen bonds (distance between O−H of the guest and O=C of CB*n* in Å): CB7 ⋅ 1‐AdOH (2.0), CB7 ⋅ FeCp_2_OH (2.0), CB8 ⋅ 4‐DAOH (2.4), and CB8 ⋅ 4,9‐DA(OH)_2_ (2×2.4).

Except for CB7 ⋅ 1‐AdOH and CB7 ⋅ FeCp_2_OH all binding free energies coincided in the relatively narrow range of Δ*G*_exp_=−9.0 to −9.9 kcal mol^−1^ despite the substantial differences in size fitting and packing coefficients (see Table 1 and Figure 3). In contrast, binding enthalpies varied much more between Δ*H*_exp_=−7.7 to −21.1 kcal mol^−1^ between those systems, peaking at Δ*H*_exp_=−19.0 kcal mol^−1^ for CB7 ⋅ 1‐AdOH and −21.1 kcal mol^−1^ for CB7 ⋅ FeCp_2_OH. The latter two values are in accordance with literature values, for example, −19.0 kcal mol^−1^ for CB7 ⋅ 1‐AdOH and −21.5 kcal mol^−1^ for CB7 ⋅ FeCp_2_OH, as reported by Inoue and Gilson with non‐desalted CB7 samples.^[11c]^ Generally speaking, the formation of CB7 ⋅ guest complexes was observed to be much more enthalpically favored than that of the corresponding CB8 ⋅ guest complexes. The results were surprising from a lock‐and‐key model perspective as one would expect to find more favorable binding enthalpies for the host with the larger cavity when examining size‐fitting guests (given all other effects being similar, larger guests and hosts are more polarizable and thus should experience stronger LD attraction). However, the results were not unexpected for experts in cucurbit[*n*]uril‐host‐guest chemistry that are accustomed to such striking trends from previous studies (even though they still find it hard to predict and rationalize them).[^2b^, ^17c^]

Entropic contributions to the binding were unfavorable for all CB7 ⋅ guest complexes as well as for CB8 ⋅ 3,9‐TA(OH)_2_ and CB8 ⋅ FeCp_2_OH (−*T*Δ*S*_exp_ up to 8.3 kcal mol^−1^ for CB7 ⋅ FeCp_2_OH). Mildly favorable binding entropies up to −*T*Δ*S*_exp_ *=*−2.3 kcal mol^−1^ were recorded for the complex formation of CB8 ⋅ 1‐AdOH and CB8 ⋅ diamondoids. It may be tempting to assign this higher entropic cost for forming CB7 than CB8 complexes to larger conformational/rotational restrictions of the more densely packed CB7 complexes, but it is also worth considering that the desolvation of the host cavities may come at a different entropic cost for CB7 and CB8. Overall, the aforementioned observation poses a considerable challenge for the development of an explanatory binding model despite the simplicity of the host‐guest model system.

### Quantum chemical modelling of the CB*n‐*guest complexes

DFT calculations based on two different computational protocols were employed to compute the association free energies of the investigated complexes in an effort to decipher the binding forces for CB*n*‐complex formation and to rationalize the experimentally observed trends. For both DFT protocols, the input structures of the host ⋅ guest complexes were generated by applying the conformer‐rotamer ensemble sampling tool (CREST)^[28]^ at the GFN2‐xTB^[29]^ and GFN‐FF^[30]^ level of theory with the implicit GBSA(H_2_O) solvation model.^[31]^ The energetically lowest‐lying conformers were then determined out of both runs. Due to the high rigidity, no conformer search was necessary for the individual host and guest molecules.

In the first protocol, the energetically lowest conformers, as well as host and guest molecules, were further optimized using the composite DFT method PBEh‐3c.^[32]^ Free energies were calculated based on the optimized geometries by utilizing a multilevel approach. High level single‐point energies were calculated with the hybrid density functional PBE0^[33]^ with a large def2‐TZVP^[34]^ basis set. The D4^[35]^ London dispersion correction was applied throughout. Solvation contributions to the free energy were calculated with COSMO‐RS,^[36]^ also including the volume work to convert an ideal gas at 1 bar to a solution of 1 mol L^−1^.

For the COSMO‐RS free energy, two single‐point calculations with BP86/TZ (one in the gas phase and one in an ideal conductor) were performed. The output of these calculations was then processed by the COSMOtherm program. Thermostatistical contributions to the free energy were calculated using PBEh‐3c and the modified RRHO scheme (mRRHO).^[37]^

In the computational approach, total free energies were calculated as sum of the electronic gas‐phase binding energy Δ*E* (single‐point energy), London dispersion contribution (Δ*E*_LD_), thermostatistical (Δ*G*_mRHHO_) and solvation (Δ*δG*_solv_) contribution according to Equation (1) and Equation (2), where the prefix Δ refers to the differences regarding the reaction host +guest→host ⋅ guest.

(1) $$\Delta G=\Delta E+\Delta \delta G{}_{\mathrm{solv}}+\Delta G{}_{\mathrm{mRRHO}}$$

(2) $$\Delta E=\Delta E{}_{\mathrm{el}}+\Delta E{}_{\mathrm{LD}}$$

The second protocol applies the recently published CENSO algorithm.^[38]^ Therein, the geometry optimizations were performed with the r^2^SCAN‐3c^[39]^ meta‐GGA functional employing the direct COSMO‐RS (DCOSMO‐RS) model,^[40]^ which enables geometry optimization in solution.

The single‐point calculations of the electronic energies were conducted at the same level of theory. Solvation contributions to the free energy at 298.15 K were computed from the r^2^SCAN‐3c/DCOSMO‐RS structures with the COSMO‐RS method. The thermostatistical contribution to the free energy was computed by single‐point Hessian (SPH) calculations within the framework of the mRRHO approximation at the GFN2‐xTB/ALPB level.^[41]^ The structures with the largest contribution to the calculated binding free energy computed with the r^2^SCAN‐3c/DCOSMO‐RS approach are shown in Figures 3 and S11, Supporting Information.

**Figure 4:**
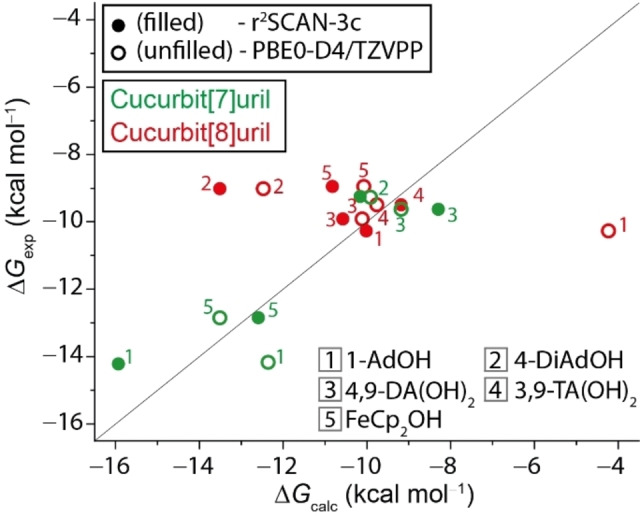
Experimental vs. calculated binding free energies (kcal mol^−1^) for the within this work investigated CB7 ⋅ guest (green) and CB8 ⋅ guest (red) complexes. Solid black line: identity. Filled symbols correlate to computationally determined values with r^2^SCAN‐3c, whereas unfilled symbols correlate to computationally determined values with PBE0‐D4/TZVPP. See Supporting Information, Table S2 for detailed values.

Comparing the two applied computational models, the biggest improvement was made for the CB8 ⋅ 1‐AdOH complex formation. When comparing the computed guest position in the CB*n* cavity, the PBE0‐D4/QZ and PBEh‐3c approaches position the hydroxy group inside the cavity without interaction with the carbonyl group, which is not realistic. The same is seen for the CB7 ⋅ 1‐AdOH complex, but due to the smaller cavity size the tilt of the adamantanol in the cavity is not as pronounced as for CB8 and therefore the differences between the calculated values with the two tested geometry generation approaches is not that large. Good correlations between theoretical and experimental binding free energy values were obtained for both computational protocols with a typical offset of ≤1.5 kcal mol^−1^ with the newly introduced r^2^SCAN‐3c model. However, large deviations between the experimental and theoretical Δ*G* values were observed for CB7 ⋅ 1‐AdOH, CB8 ⋅ FeCp_2_OH (offset 1.7 and 1.8 kcal mol^−1^), and particularly CB8 ⋅ 4‐DAOH (offset 4.4 kcal mol^−1^) even with the state‐of‐the art r^2^SCAN‐3c model. Note that deformation effects of the host molecules may account for some of the deviations, especially when comparing values of the larger‐in‐size‐host CB8. However, they cannot explain the observed trends as we would otherwise not see a difference between the two methods. A graphical summary of the data obtained by ITC in comparison to the computationally acquired values is given in Figure 4. The computed and experimental Δ*G* values for all investigated systems are given in Supporting Information, Table S2.

## Discussion

In the following, we first interpret the experimental thermodynamic parameters followed by a discussion of the computationally determined energies in order to shed light on the specific binding mode of CB7 and CB8 with the diamondoid alcohols, and for CB*n* complexes in general.

**Figure 5:**
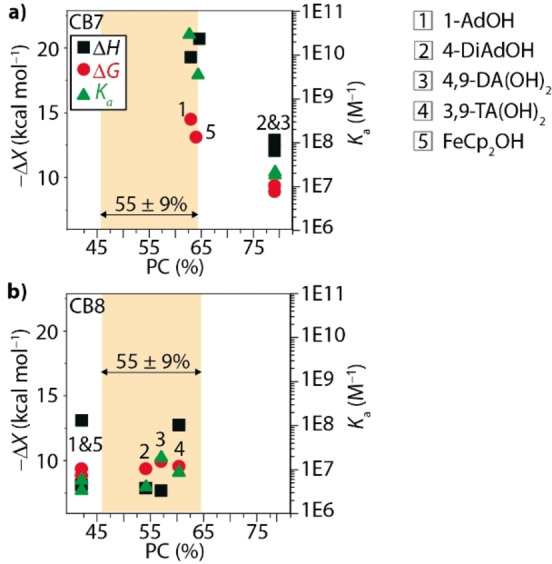
Packing coefficients (PCs) vs. experimentally determined thermodynamic parameters (Δ*H*_exp_=black squares, Δ*G*_exp_=red circles) of the investigated a) CB7 ⋅ guest complexes and b) CB8 ⋅ guest complexes. The PC of 55±9 % for an optimal binding^[42]^ is marked in yellow. Furthermore, the binding affinity *K*_a_ (green triangles) is correlated to the PCs. For PC calculations, only atoms located inside of the cavity were considered, see Table S1, Supporting Information for details.

When inspecting the plot of the binding parameters *K*_a_, Δ*G*_exp_, and Δ*H*_exp_ vs. the packing coefficients (PCs) of the different CB7/CB8 ⋅ amantane complexes (Figure 5), it is immediately clear that there is no general predictive power of the Rebek and Mecozzi 55±9% rule for these model systems.^[42]^ While the ordering amongst the CB7 complexes seems to clearly correlate with the packing coefficient argument, even the CB7 complexes with a remarkably high and supposedly unfavorable PC of 79 % still possess a similar binding affinity and binding enthalpy as supposedly optimally spatially fitting complexes of CB8. For comparison, it was originally proposed that host‐guest complexes with a PC ∼70 % require stabilization by strong intermolecular forces such as hydrogen bonds, while a PC of ∼80 % completely prevented host‐guest complexation for molecular capsules.^[42]^ Likewise, the binding free energy differences amongst the CB8 complexes with optimal or too low/too high PC values is also only modest. Thus, the 55±9% rule may be used to rationalize the expected binding differences for a given host (e. g., CB7), but there seem more important factors at play that determine the differences between the host homologues CB7 and CB8. This finding is difficult to explain from a point of view that focuses on the direct binding interactions between host and guest, for example, the lock‐and‐key model.

**Figure 6:**
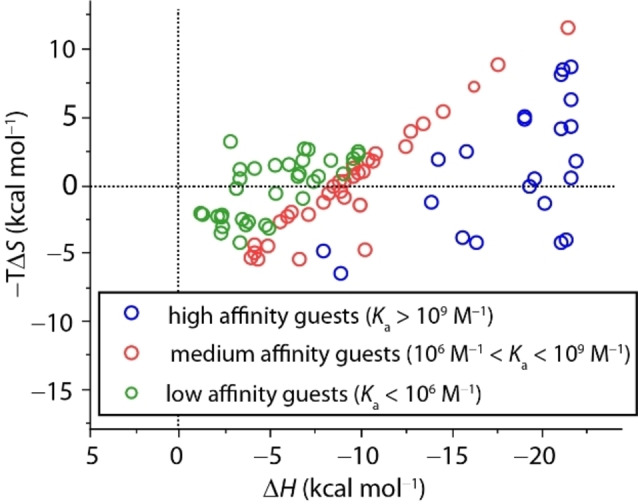
Correlation plot between entropic (−*T*Δ*S*_exp_) and enthalpic (Δ*H*_exp_) contribution to the overall free association energy Δ*G*_exp_ for CB7 with high (*K*_a_>10^9^ M^−1^, blue), medium (10^6^ M^−1^<*K*_a_<10^9^ M^−1^, red), and low (*K*_a_<10^6^ M^−1^, green) affinity binders at 25±5 °C in solution with up to 10 mM salt content (detailed information about the data origin/data sets is given in the Supporting Information, Table S5).

As CB7 is exceptional with respect to affinity and binding enthalpy amongst all known artificial receptors, we conducted a literature survey for CB7 complexes for which experimental Δ*G*_exp_, Δ*H*_exp_, and *T*Δ*S*_exp_ binding contributions are known. The resulting correlation graph of a set of 21 high‐affinity guests (*K*_a_>10^9^ M^−1^), 33 medium‐affinity guests (10^6^ M^−1^<*K*_a_<10^9^ M^−1^), and 32 relatively low affinity guests (*K*_a_<10^6^ M^−1^) is shown in Figure 6.

Firstly, all CB7 complex formations are exothermic and span a wide range from −1 to −22 kcal mol^−1^. Generally speaking, weak binders (*K*_a_<10^6^ M^−1^) do not reach enthalpic contributions higher than Δ*H*∼−10 kcal mol^−1^ whereas the complex formation of strong CB7 ⋅ guest complexes (*K*_a_>10^9^ M^−1^) are highly enthalpically favored. Strikingly, there appears to be a ceiling of the maximum Δ*H* contribution of about −22 kcal mol^−1^. Such a behavior is difficult to rationalize if direct binding interactions such as LD or electronic contributions dominated the binding process whereas a host‐dominated enthalpic driving force, for example, through a maximum high‐energy cavity water release, remains a conceivable model.

**Figure 7:**
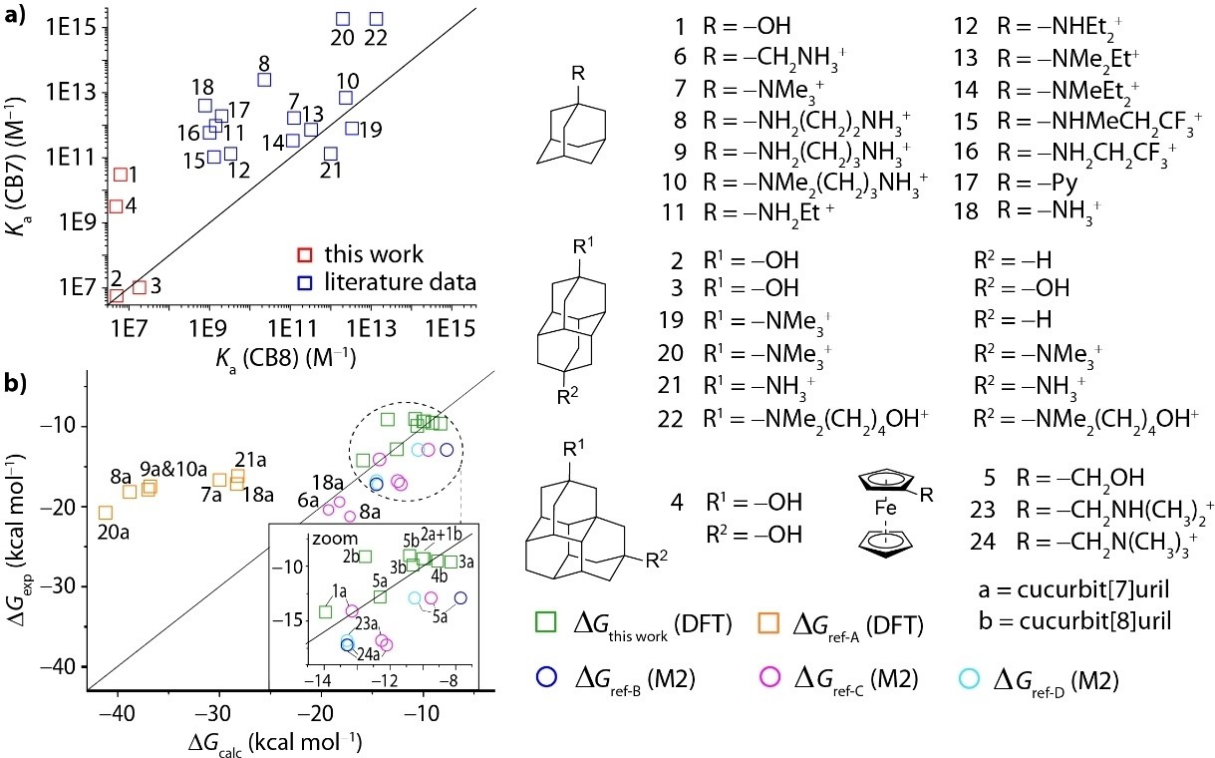
a) Comparison of binding affinities of CB7 and CB8 for several adamantane‐ and ferrocenyl‐derivatives determined in water. b) Comparison of experimentally determined Δ*G*_exp_ and computed total free association energies Δ*G*_calc_ obtained by either DFT (this work and ref‐A^[46]^) or M2 (ref‐B^[47]^, ref‐C^[11c]^, ref‐D^[3]^) calculations. All energy values were referenced to 1 mol L^−1^. See Supporting Information, Section 7, for details on the energy conversion from the 1‐atm standard state to the 1‐M standard state for the computed binding free energies, and Tables S2–Sr, and Figure S12.

The entropic contributions, −*T*Δ*S*, for CB7 complex formation span a narrower range from unfavorable −7 to favorable 11 kcal mol^−1^, where most examples fall within −5 to 5 kcal mol^−1^. Noteworthy, there is no indication for the usually dreaded enthalpy‐entropy compensation amongst the high‐affinity guests.^[43]^ If the entropic contribution is further optimized while the large binding enthalpy is maintained, it may be therefore possible to find guests with even higher affinities than the dicationic diamantanes[^11c^, ^44^] reported by Isaacs and coworkers. An overview of some of the strongest reported binders for CB7 and CB8, mostly positively charged diamondoids and ferrocenes, is shown in Figure 7. Again, the binding affinities are (much) larger for their CB7 than for their CB8 complexes for all but three compounds. Even the recently reported dye methyl‐pyridinium‐paracyclophane (MPCP), a cationic and rigid guest which is snug fitting inside the CB8 cavity and too large for the CB7 cavity, “only” has a binding affinity of *K*_a_=4×10^12^ M^−1^ for CB8 in deionized water.^[25b]^ In general, one would expect that when considering similar in size fitting guests towards CB7 and CB8, the CB8 ⋅ guest complex shows the stronger binding affinity due to the larger molecular size and higher polarizability.

As the investigated guests differ widely in terms of size dimension, and thus in the packing coefficients for the CB*n*‐guest complexes, the observed clear trend points to some special CB7‐specific reason for its role as an ultra‐high affinity binder. If host‐guest interactions were the dominating factor, surely also some of the CB8 ⋅ guest complexes would excel in affinity when their packing coefficients and other direct host‐guest bonding interactions are superior to that of the corresponding CB7 complex. For comparison, both β‐cyclodextrin and γ‐cyclodextrin, which have cavity dimensions similar to that of CB7 and CB8, respectively, show peaking affinities when the guest optimally fits to either of the hosts.^[45]^ Moreover, the larger host‐guest system formed by γ‐cyclodextrin with dodecaborate clusters, is even bound more strongly and is more exothermic than that of the corresponding gold‐standard β‐cyclodextrin complexes with its optimally fitting adamantyl‐ or ferrocenyl‐type guests. The special role of the smaller host CB7 is therefore not following the usual supramolecular concepts.

Our study on uncharged diamantanes also reveals that Isaacs’ ultra‐high affinity CB7 complexes with dicationic diamantanes[^11c^, ^44^] are likely not benefiting from some particularly attractive interactions between the CB7 host and the diamantane scaffold. In contrast, this direct interaction is even less attractive than that of adamantane, as can be deduced from the less favorable binding free energy and much less favorable binding enthalpy of the CB7 complexes with 4‐DAOH and 4,9‐DA(OH)_2_ compared to that with 1‐AdOH. Possibly, there is a repulsive contribution for threading the diamantane scaffold through the carbonyl‐fringed, polar, and well solvated portal regions of the CB7 host. Thus, the beneficial role of the diamantane scaffold in Isaacs’ guest design may be attributed to providing the perfect distance between the two ‐NMe_3_^+^ moieties such that they can optimally engage through cation‐π interactions with both carbonyl‐fringed portal regions of CB7 simultaneously.[^3^, ^44a^] Consequently, it may be promising to investigate other organic scaffolds that are less repulsive towards CB7 than diamantanes but still provide an ideal size fit and enable perfect positioning of positively charged moieties.

**Figure 8:**
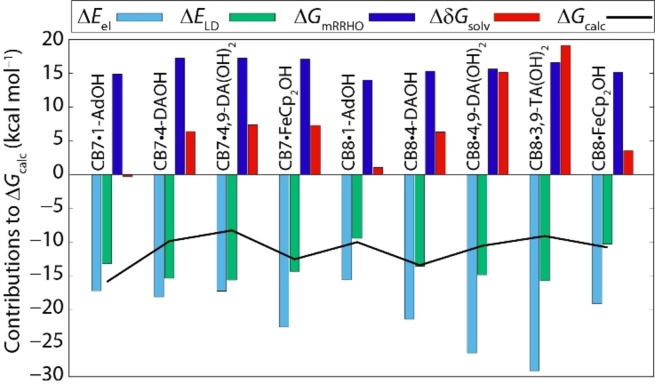
Contributions to Δ*G*_calc_ with electronic energy (Δ*E*_el_), LD energy (Δ*E*_LD_), thermal corrections from energy to free energy (Δ*G*_mRRHO_), and solvation free energy (Δ*δG*_solv_) for the r^2^SCAN‐3c method. All values are given in kcal mol^−1^.

Despite some successes for the computational determination of binding affinities of CB*n*‐guest complexes with simple guests,^[14b]^ there are still many challenges. For instance, a systematic study on cationic diamondoids as guests for CB7 and CB8 yielded binding energy mean discrepancies of 3.2 kcal mol^−1^ (maximum discrepancy 6.7 kcal mol^−1^) between the experimental and computed (BLYP‐D3/def2‐TZVPP) Δ*G* values with theory largely overestimating the binding affinities (factor 10^1^–10^5^), see also Figure 7(b).^[44b]^ A better agreement between theory and computations was found for uncharged hydrocarbon guests with CB7 at the DFT level (RSME of 1–2 kcal mol^−1^).^[17a]^ Nevertheless, the QM4 method yielded substantial overbinding (4 kcal mol^−1^) for adamantane. Force‐field based MD computations also resulted in large overbinding discrepancies (RSME of 5.5 kcal mol^−1^) for the hydrocarbon guest set.^[17a]^ In comparison, the agreement between experimentally measured and computationally determined binding free energies is much better with the herein reported r^2^SCAN‐3c model and allows for meaningful mechanistic analysis of the driving forces for host‐guest complexation. Figure 8 displays the energy decomposition analysis for the geometry optimized CB7 and CB8 complexes with the diamondoid guests. Several conclusions can be drawn from this data set: Firstly, the computed LD energies of the host‐guest complexes are rather constant Δ*E*_LD_=−14.7±1.0 kcal mol^−1^) across the different host‐guest systems (except for CB8 ⋅ AdOH and CB8 ⋅ FeCp_2_OH, see also Supporting Information, Table S3), suggesting that LD does not play a general role in defining the binding preferences of CB*n* for different guests. Nevertheless, markedly lower LD energy contributions for CB8 ⋅ AdOH (−9.5 kcal mol^−1^) and for CB8 ⋅ FeCp_2_OH (−10.3 kcal mol^−1^) are attributable to the poor fit for these small guests inside the large cavity of CB8 and this may be one of the reasons for their comparably low binding enthalpy and binding free energy. However, LD energies cannot explain the generally observed much larger binding enthalpies of CB7 complexes compared to CB8 complexes with size fitting guests.

There are modest variations in the computed entropic contributions (mRRHO model) to binding for the different host‐guest complexes which were generally unfavorable. Specifically, the computed Δ*G*_mRRHO_ is more unfavorable for CB7 than for CB8 complexes, Δ*G*_mRRHO_ = and 15.0 kcal mol^−1^, respectively, which is in line with experimentally seen larger entropic cost for forming CB7 than CB8 complexes −*T*Δ*S*_exp_=4.7 versus 1.6 kcal mol^−1^). However, entropic effects do not dominate the overall binding characteristics for the herein presented guests as CB7 is at least as strongly binding as CB8.

Much larger guest and host dependent variations are found for the electronic energy contributions (Δ*E*_el_) of the host‐guest complexes but it is difficult to intuitively rationalize these energy differences by the binding geometry of the host‐guest complex and electrostatic potential plots (Figure 3). For instance, the spatial proximity and/or number of hydrogen‐bonding interactions between the diamondoids and CB8 correlates in some cases to the electronic binding energy as in the series 1‐AdOH (no H‐bond; Δ*E*_el_=−15.6 kcal mol^−1^), 4‐DAOH (d_OH⋅⋅⋅O=C_=2.4 Å; Δ*E*_el_=−21.5 kcal mol^−1^), 4,9‐DA(OH)_2_ (2× d_OH⋅⋅⋅O=C_=2.3 Å; Δ*E*_el_=−26.5 kcal mol^−1^) but the CB8 complex with 3,9‐TA(OH)_2_ (no H‐bond; Δ*E*_el_=−29.1 kcal mol^−1^) does not follow the trend. Unlike for CB8, the electronic energy contributions of the CB7 ⋅ guest complexes are rather similar to each other (Δ*E*_el_=−17.5±0.5 kcal mol^−1^) despite their notable differences in the H‐bonding pattern: 1‐AdOH (*d*_OH⋅⋅⋅O=C_=2.0 Å), 4‐DAOH (no H‐bond), and 4,9‐DA(OH)_2_ (no H‐bond).

It is important to mention that the mean electronic contributions to binding ($\overset{-}{\Delta E_{\mathrm{el}}}$ =−18.8 and −20.7 kcal mol^−1^ for CB7 and CB8, respectively) do not explain the much more exothermic mean binding enthalpy of the herein reported CB7 complexes ($\overset{-}{\Delta H_{\exp}}$ =−16.2 kcal mol^−1^) versus the corresponding CB8 complexes ($\overset{-}{\Delta H_{\exp}}$ =−9.2 kcal mol^−1^) with the same guests, even if one removes the small‐sized, excellent CB7‐binders AdOH and FeCp_2_OH from the comparison. Clearly, counterbalancing (de)solvation effects need to be factored in, see below.

The so far discussed computed binding contributions, for example, electronic and LD energy and entropy, do not reproduce the experimental binding trends within the guest series, and particularly not between CB7 and CB8. Thus, it can be deduced that the solvation free energy differences between the host‐guest complexes, and between the unbound host and guest must play a decisive role.

Solvation energies are notoriously difficult to experimentally measure for anything but small and volatile molecules.^[48]^ Hence, experimental values for host molecules with concave cavities such as cucurbit[*n*]urils are completely absent from the training sets, such that it remains to be carefully considered how implicit solvation models^[49]^ perform for such systems. Be this as it may, the herein employed continuum solvation COSMO model[^36b^, ^40^, ^50^] family has proven useful in the past for several systems, including host‐guest complexes.^[51]^

The computed solvation free energies (Δδ*G*_solv_) for the whole process, i. e., host_aq._+guest_aq._→[host ⋅ guest]_aq._, were mostly in agreement with an intuitive binding model. For instance, the solvation energy contributions are very similar for CB7 ⋅ 4‐DAOH (6.4 kcal mol^−1^) and CB7 ⋅ 4,9‐DA(OH)_2_ (7.4 kcal mol^−1^), which is consistent with the computed binding geometry where the hydroxylgroups are pointing outwards of the host‐guest complex. For comparison, Δδ*G*_solv_ became much more unfavorable for the CB8 complexes in the series of 1‐AdOH (1.0), 4‐DAOH (6.3), 4,9‐DA(OH)_2_ (15.2) to 3,9‐TA(OH)_2_ (19.1 kcal mol^−1^), thereby counterbalancing the series trend of the aforementioned stabilizing Δ*E*_el_ contributions. This can be understood by the buried and thus desolvated OH‐groups for the CB8 complexes with the diamondoids (Figure 3).

Nevertheless, the COSMO‐RS model also produced results that were surprising, for instance the computed solvation free energies of 1‐AdOH (−4.0), 4‐DAOH (−4.9), 4,9‐DA(OH)_2_ (−10.5) to 3,9‐TA(OH)_2_ (−10.4 kcal mol^−1^). While it is perfectly intuitive that compounds with two OH‐groups are better solvated than the monohydroxylated guests, our chemical intuition, the solubilities of diamondoids, and a force‐field study^[52]^ suggested that the solvation free energies of the diamondoids should become much less favorable in the order of adamantane>diamantane>triamantane. The herein utilized COSMO‐RS model did not reproduce this expected trend for the solvated guests at all. The COSMO‐RS based solvation free energies did also not provide a clear‐cut rational for the strong experimental binding enthalpy differences between CB7 and CB8 complexes, regardless of the aforementioned necessary precautions for applying implicit solvent models to concave hosts. Tentatively, we assign the major part of the remaining errors of our quantum chemical binding free energies compared to experiment to the solvation contribution while practically non‐empirical electronic, dispersion as well as mRRHO contributions together seem to have a smaller error. One reason for this is probably that the empirical elements in the COSMO‐RS model have been determined mainly for relatively small molecules (mostly less than 20–30 atoms) while the here considered complexes have more than 100 atoms.

## Conclusion

The pursued careful calorimetric investigation and joined theoretical modelling of nine high affinity host ⋅ guest complexes of CB7 and CB8 with spherically shaped, conformationally rigid, and uncharged hydrophobic guests provides deep insights into the molecular binding mechanism of the remarkably strongly guest‐binding cucurbit[*n*]uril macrocyclic family. The discussed structure‐property trends and a comparison to literature reports clearly rule out direct host‐guest binding interactions as the decisive factor for explaining the guest‐binding selectivity. They also cannot rationalize the uniquely strong and exothermic binding characteristics of CB7. Both London dispersion interactions and electronic energies did not correlate to the experimentally seen trends. Entropic factors do contribute significantly to the overall free binding energies but are also not a major factor. Likewise, empirical supramolecular concepts such as Rebek's and Mecozzi's 55 % rule^[42]^ are only of modest predictive utility. Indeed, the binding enthalpy of hydroxylated diamantanes is still much more exothermic for CB7 than for CB8, despite the remarkably high PC of 79 % for the CB7 complex versus the near “optimal” 54 and 57 % PCs for the corresponding CB8 complexes. These findings are likely transferable to other aqueous host‐guest systems where the binding geometry is relatively compact and the host‐bound guest is shielded from solvent contact.

Furthermore, it was shown in this study that the recently introduced r^2^SCAN‐3c quantum chemical model coupled with extensive complex conformer searches performs superior over the previous PBEh‐3c composite method for determining the binding energies of the herein investigated high‐affinity complexes of CB7 and CB8 with uncharged, spherically shaped, and conformationally rigid guests. Nevertheless, the computed solvation free energies of the diamondoids were in part in conflict with chemical intuition.

Overall, this study provides a clear indication that peculiar host‐related solvation effects are likely the major factor for rationalizing the unique behavior and record‐affinity characteristics of cucurbit[*n*]urils. Further research for improving both the experimental and theoretical toolbox for assessing the solvation free energies of concave cavity hosts and other synthetic receptors is well justified as it will allow to assess more rigorously contemporary aqueous binding models of supramolecular systems.

## Experimental Section

**Materials**: All commercial chemicals used in this study were used without further purification. Cucurbit[*n*]urils were purchased from Strem Chemicals or synthesised following the published path^[53]^ and desalinated with a biotech grade dialysis membrane (cellulose ester/regenerated cellulose) system. Diamantane‐ and triamantane‐ hydroxy derivatives were synthesized following literature procedures.^[23]^

**NMR spectroscopy**: ^1^H NMR spectra were recorded either in deuterium oxide or in a mixture of deuterium oxide and MeOH‐*d_3_* on a Bruker Avance 500 spectrometer at 25 °C. The ^1^H NMR chemical shifts (δ) are given in ppm and refer to residual protons of the corresponding deuterated solvent.

**Isothermal titration calorimetry**: ITC experiments were carried out on a Microcal PEAQ‐ITC from Malvern. All experiments were conducted under air in Millipore H_2_O at 25 °C. In a typical experiment, 1.5 μL guest solution (the first injection was 0.4 μL) were injected 25 times into the ITC cell (spacing: 150 s; stir speed: 750 rpm; initial delay: 60 s; injection duration: 6 s), which contained the host at a 10 times lower concentration. If not stated otherwise, the data was baseline corrected by the average value of the titration curve of guest into water. Typical errors, determined by repeating the titrations at least three times, are 20 % in *K*_a_, 0.2 in log *K*_a_, 0.4 kcal mol^−1^ in Δ*H*_exp_ and Δ*G*_exp_, and 0.8 kcal mol^−1^ in −*T*Δ*S*_exp_. The data was analyzed by Microcal PEAQ‐ITC analysis software with the one‐set‐of‐sites model, and the first data point from the 0.4 μL injection was always omitted.

**UV‐Vis spectroscopy**: Absorption spectra were measured on a Jasco V‐730 double‐beam UV‐Vis spectrophotometer and baseline corrected.

**Fluorescence spectroscopy**: Steady‐state emission spectra were recorded on a Jasco FP‐8300 fluorescence spectrometer equipped with a 450 W xenon arc lamp, double‐grating excitation and emission monochromators. Emission and excitation spectra were corrected for source intensity (lamp and grating) and the emission spectral response (detector and grating) by standard correction curves. Fluorescence‐based titration curves were performed by an ATS‐827 automatic titration unit filled with the appropriate guest to obtain the *K*_a_ values in case of CB7 ⋅ BC, CB7 ⋅ MDAP, and CB7 ⋅ AdOH complex. The acquired data was then fitted following the fitting equations listed in Supporting Information, Section 4. All binding affinity measurements were repeated at least three times for all systems studied and the typical errors were determined to be less than 20 % in *K*_a_ and less than 0.2 in log *K*_a_.

**Quantum chemical calculations**: All quantum mechanical calculations were performed with TURBOMOLE 7.2.1^[54]^ (DFT) and xTB 6.3.2^[55]^ (GFN1‐xTB, GFN2‐xTB, GFN‐FF) program packages with default convergence criteria 10^−7^ *E*_h_ for energies and 10^−5^ *E*_h_ *Bohr* ^−*1*^ for gradients. The resolution‐of‐identity (RI) approximation for the Coulomb integrals was generally used to speed up the DFT calculations using matching default auxiliary basis sets.^[56]^ For the integration of the exchange‐correlation contribution, the numerical quadrature grid *m4* was employed. All calculations were performed on Intel^©^ Xeon E5‐2660 v4 @ 2.00 GHz machines. Conformers were generated with the conformer‐rotamer sampling tool CREST.^[28]^

## Conflict of interest

The authors declare no conflict of interest.

1

## Supporting information

As a service to our authors and readers, this journal provides supporting information supplied by the authors. Such materials are peer reviewed and may be re‐organized for online delivery, but are not copy‐edited or typeset. Technical support issues arising from supporting information (other than missing files) should be addressed to the authors.

Supporting Information

## Data Availability

All data are available from the corresponding authors upon reasonable request and are digitally stored on the servers of the home institute. Furthermore, binding parameters can be found on suprabank.org; https://doi.org/10.34804/supra.20220323420.
