# Supplementary material for: The Role of Packing, Dispersion, Electrostatics, and Solvation in High‐Affinity Complexes of Cucurbit[n]urils with Uncharged Polar Guests
Source: Chemistry. 2022 May 25;28(38):e202200529. doi: 10.1002/chem.202200529 (PMC9401061; doi:10.1002/chem.202200529)
Supplement: Supplementary file 1 — Supporting Information [file CHEM-28-0-s001.pdf]

Chemistry–A European Journal

Supporting Information

The Role of Packing, Dispersion, Electrostatics, and Solvation in High-Affinity Complexes of Cucurbit[*n*]urils with Uncharged Polar Guests

Laura M. Grimm, Sebastian Spicher, Boryslav Tkachenko, Peter R. Schreiner,*
Stefan Grimme,* and Frank Biedermann*

1. Sample preparation

All stock solutions were prepared in Millipore H_2O and kept in the fridge at +4 °C for storage. Concentrations of dye stock solutions were determined by UV-Vis absorption titration measurements (BC: $\varepsilon_{(344nm)}$ = 22300 $M^{-1}cm^{-1}$;[1] cobaltocene$^+$: $\varepsilon_{(261nm)}$ = 34200 $M^{-1}cm^{-1}$;[2] MDAP: $\varepsilon_{(393nm)}$ = 7800 $M^{-1}cm^{-1}$; MPCP: $\varepsilon_{(335nm)}$ = 7111 $M^{-1}cm^{-1}$ [3]). Concentrations of CB7 and CB8 solutions were determined by ITC titration experiments with bis(cyclopentadienyl)cobalt(III)hexafluorophosphate as guest and by independent emission-based IDAs with MDAP (CB7) and MPCP (CB8). The determined concentrations of the two different methods were in good agreement. The concentration of the guest molecules studied were determined by ITC titrations with known concentrations of either CB7 or CB8 or in the case of 1-AdOH by IDA titration with MDAP.

2. NMR binding experiments

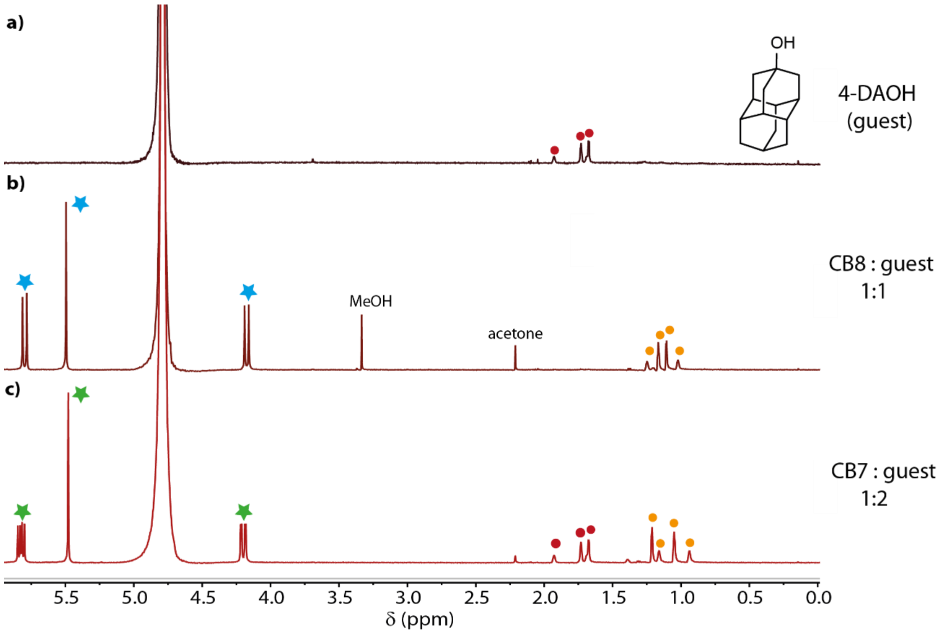

Figure S 1. ^{1}H NMR spectra (500 MHz, D_2O, r.t.) recorded for a) 4-DAOH (c = 250 µM), b) CB8•4-DAOH (c = 250 µM each), and c) a mixture of CB7 and 4-DAOH (c(CB7) = 250 µM and c(4-DAOH) = 500 µM). Free guest = red dot; bound guest = orange dot; CB7 = green star; CB8 = blue star.

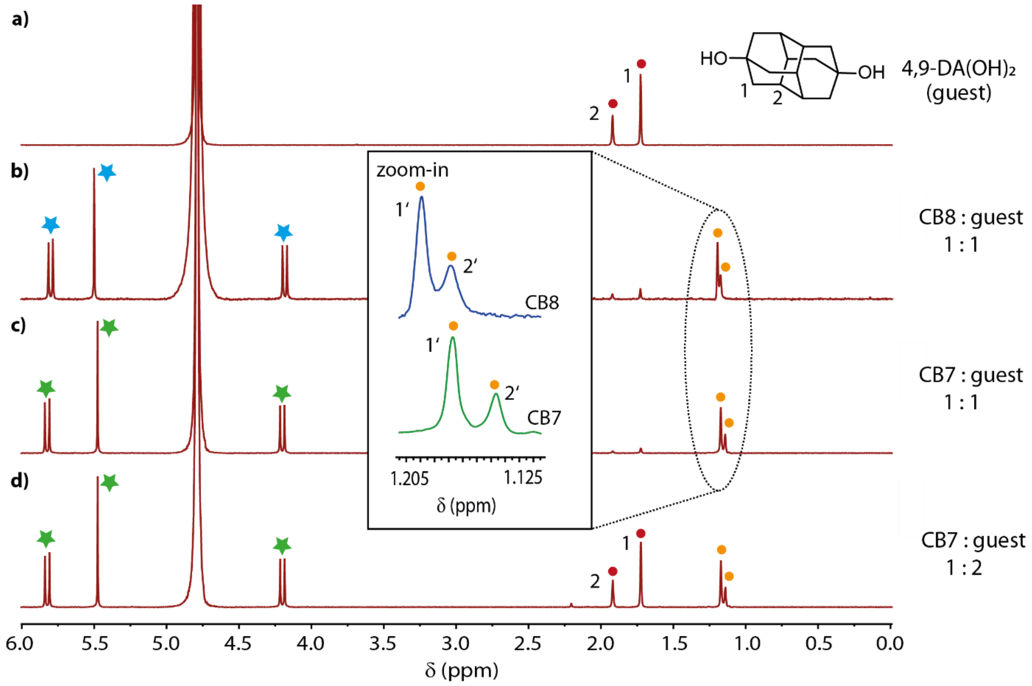

Figure S 2. ^{1}H NMR spectra (500 MHz, D_2O, r.t.) recorded for a) 4,9-DA$(OH)_2$ (c = 250 µM), b) CB8•4,9-DA$(OH)_2$ (c = 250 µM each), c) CB7•4,9-DA$(OH)_2$ (c = 250 µM each), and d) a mixture of CB7 and 4,9-DA$(OH)_2$ (c(CB7) = 250 µM and c(4,9-DA$(OH)_2$) = 500 µM). Free guest = red dot; bound guest = orange dot; CB7 = green star; CB8 = blue star.

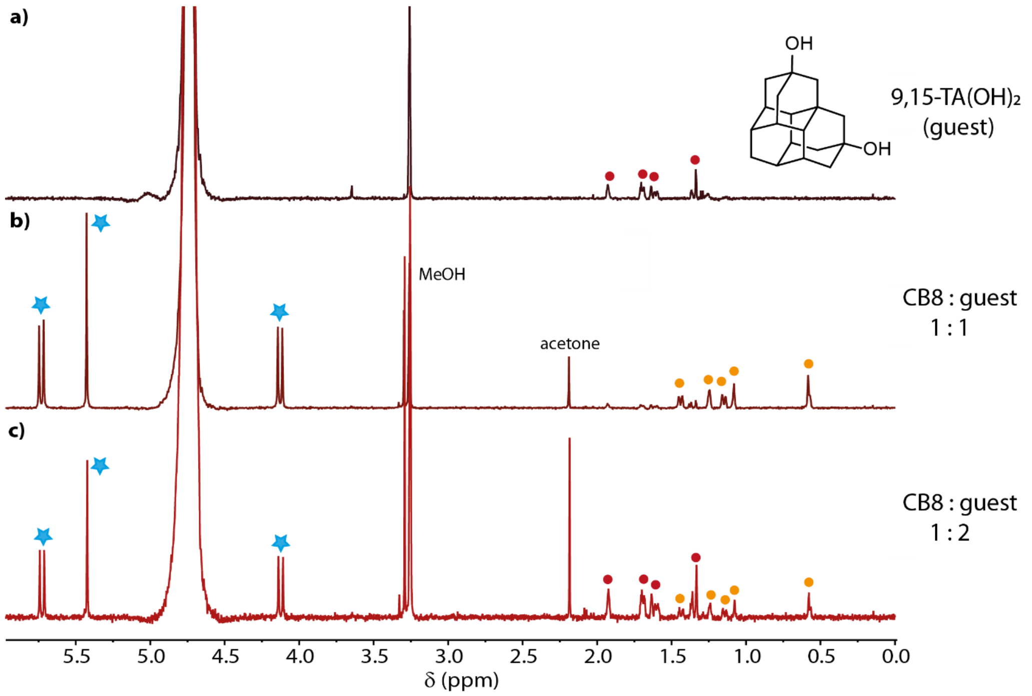

Figure S 3. ^{1}H NMR spectra (500 MHz, D_2O + 10% MeOH/MeOD-d_3, r.t.) recorded for a) 9,15-TA$(OH)_2$ (c = 250 µM), b) CB8•9,15-TA$(OH)_2$ (c = 250 µM each), and c) a mixture of CB8 and 9,15-TA$(OH)_2$ (c(CB8) = 250 µM and c(9,15-TA$(OH)_2$) = 500 µM). Free guest = red dot; bound guest = orange dot; CB8 = blue star.

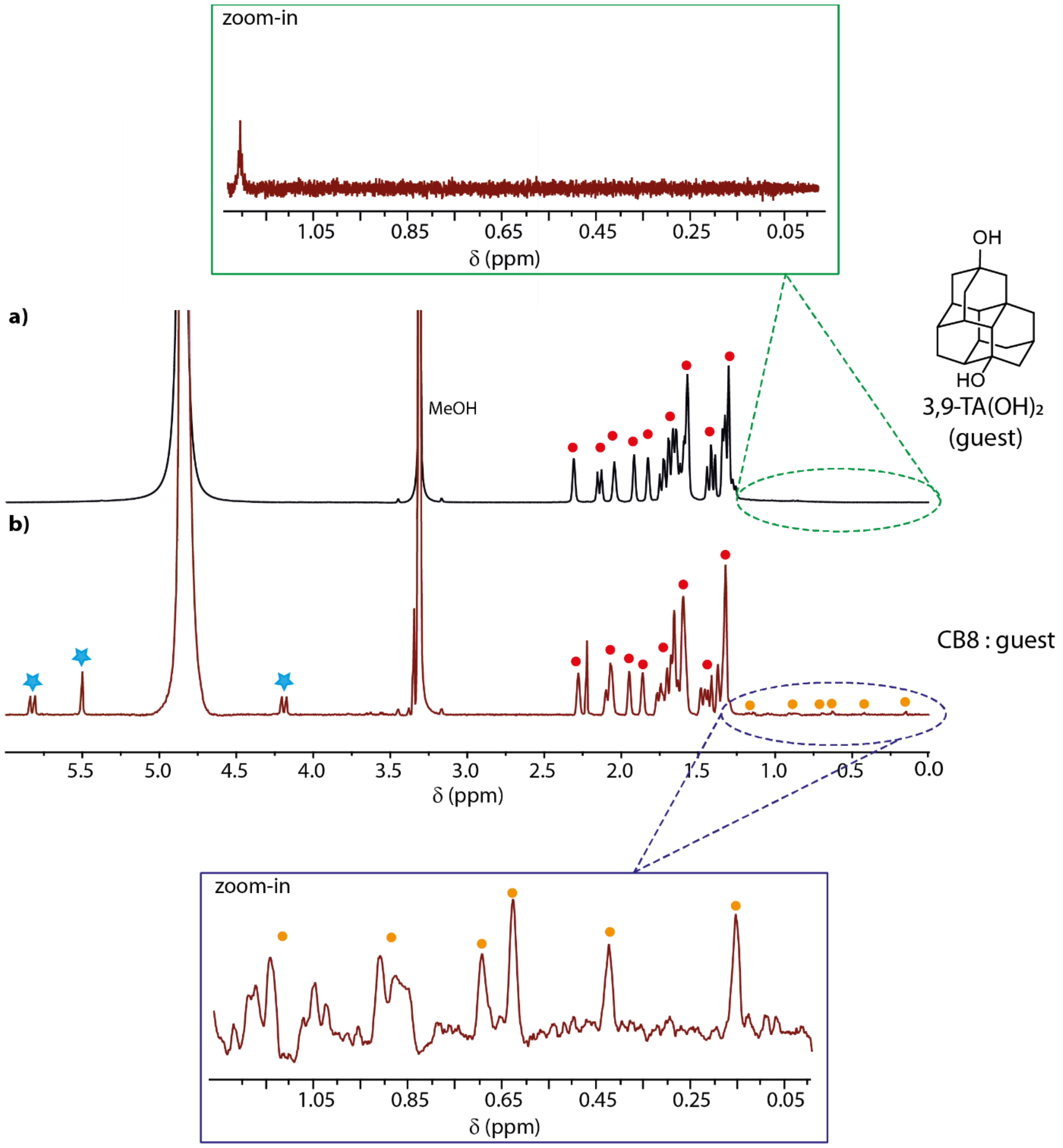

Figure S 4. ^{1}H NMR spectra (500 MHz, D_2O + 20% MeOH/MeOD-*d*$_3$, r.t.) recorded for a) 3,9-TA$(OH)_2$ (c = 250 µM), and b) a mixture of CB8 and 3,9-TA$(OH)_2$. Free guest = red dot; bound guest = orange dot; CB8 = blue star. We found that mixing of a solution of CB8 and 3,9-TA$(OH)_2$ led to precipitation of a white solid, presumably of the CB8•3,9-TA$(OH)_2$ complex. However, the remaining small NMR peaks visible in the zoom-in were assignable to the residual amount of complex of CB8•3,9-TA$(OH)_2$ dissolved in D_2O. The clear upfield shift of the triamantane protons confirm that also for CB8•3,9-TA$(OH)_2$ an inclusion complex binding geometry is formed, which is also in accordance with the computational determinations shown in Manuscript Figure 3 and Figure S11.

3. ITC experiments

3.1. Reproducibility

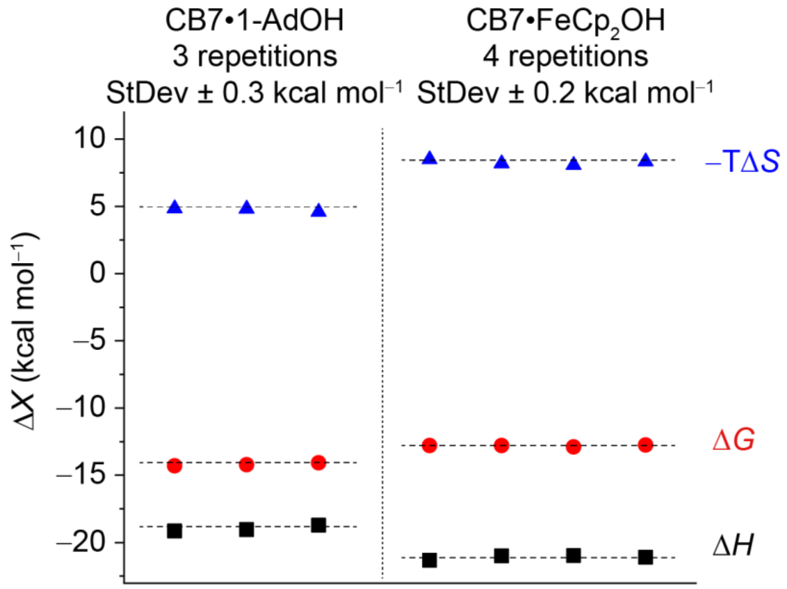

Figure S 5. Individually measured repetitions and standard deviation (StDev) of the determined thermodynamic binding parameters ΔH (black square), ΔG (red dot), and $-T\Delta S$ (blue triangle) exemplarily shown for the complex formation of CB7•1-AdOH and CB7•$FeCp_2OH$.

3.2. 1-Adamantanol - 1-AdOH

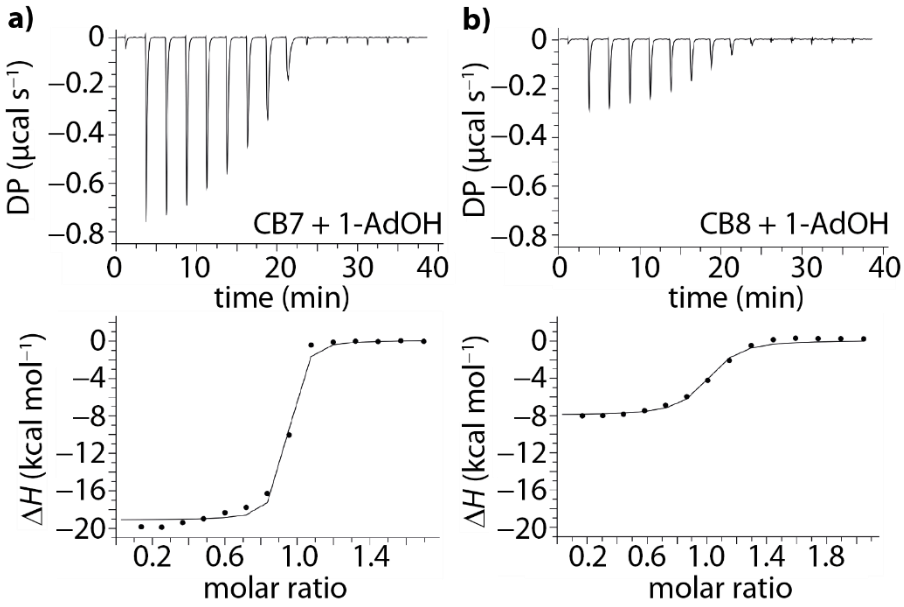

Figure S 6. ITC isotherms (dilution heat corrected) for the titration of 1-AdOH (c = 0 - 35 µM) to a) CB7 (c = 19 µM) and b) CB8 (c = 16 µM) in water at 25 °C.

3.3. 4-Hydroxydiamantane - 4-DAOH

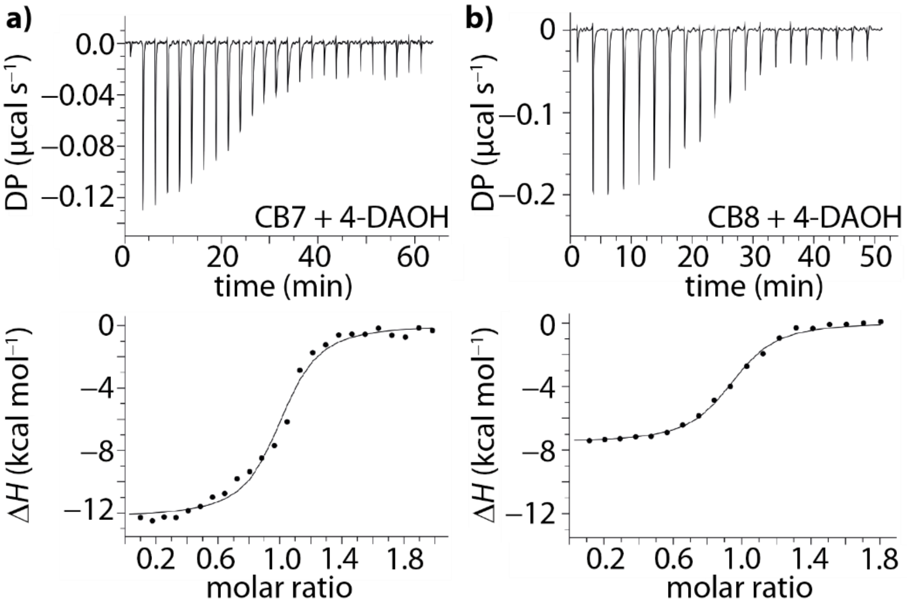

Figure S 7. ITC isotherms (dilution heat corrected) for the titration of 4-DAOH (c = 0 - 20 µM) to a) CB7 (c = 8 µM) and b) CB8 (c = 11 µM) in water at 25 °C.

3.4. 3,9-Dihydroxytriamantane - 3,9-TA(OH)$_2$

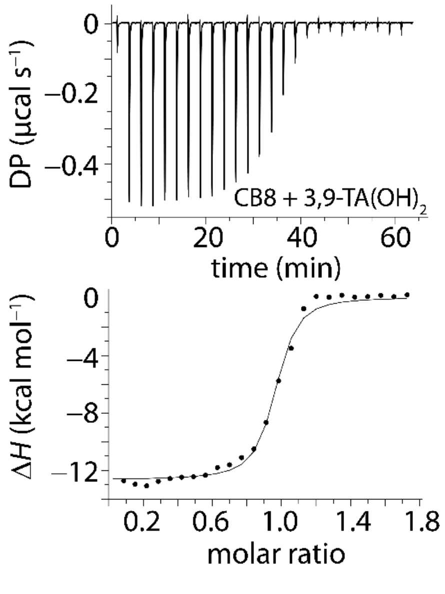

Figure S 8. ITC isotherm (dilution heat corrected) for the titration of 3,9-TA(OH)$_2$ (c = 0 - 40 µM) to CB8 (c = 22 µM) in water at 25 °C.

3.5. Ferrocenylmethanol - $FeCp_2OH$

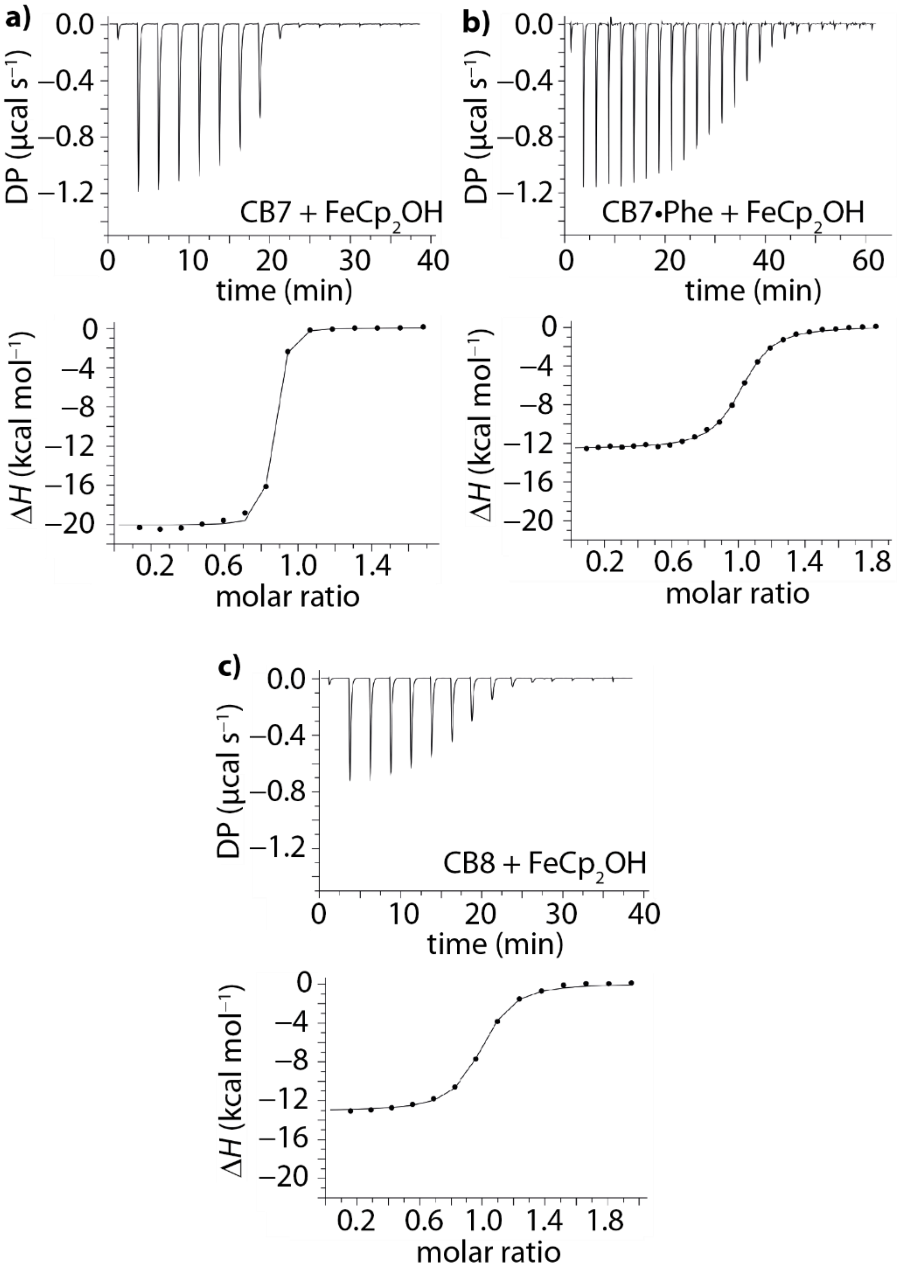

Figure S 9. ITC isotherms (dilution heat corrected) for the titration of a) $FeCp_2OH$ (c = 0 - 55 µM) to CB7 (c = 30 µM), b) $FeCp_2OH$ (c = 0 - 100 µM) to a mixture of CB7 (c = 63 µM) and Phe (c = 1.5 mM),[4] and c) $FeCp_2OH$ (c = 0 - 55 µM) to CB8 (c = 26 µM) in water at 25 °C.

4. Emission-based binding affinity determination

The binding affinities of BC, MDAP, and 1-AdOH towards CB7 were determined by fluorescence-based titration experiments. For BC, a binding affinity to CB7 of log K_a = 7.0 was determined with a direct binding assay (λ_{ex} = 440 nm, λ_{em} = 542 nm). For MDAP, an indicator displacement assay (λ_{ex} = 421 nm, λ_{em} = 542 nm) with BC as competitor was assessed and a binding affinity to CB7 of log K_a = 9.0 was determined. For 1-AdOH, a similar IDA assay was used but with MDAP as indicator (λ_{ex} = 378 nm, λ_{em} = 427 nm, log K_a = 10.4). The reaction times for each titration step were determined by kinetic investigation.

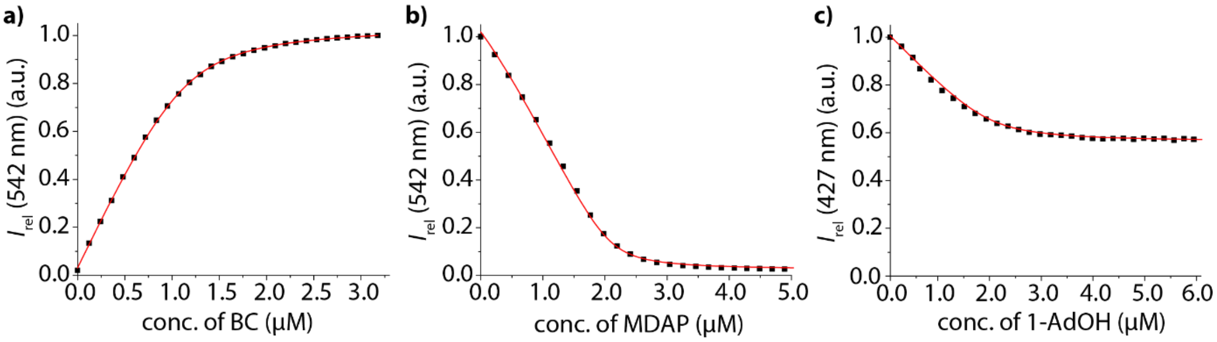

Figure S 10. a) Fluorescence intensity at 542 nm (λ_{ex} = 440 nm) after the attainment of the equilibrium (30 s reaction time) as a function of BC concentration at 25 °C; c(CB7) = 1.15 µM. b) Fluorescence intensity at 542 nm (λ_{ex} = 421 nm) after the attainment of the equilibrium (15 s reaction time) as a function of MDAP concentration at 25 °C; c(CB7) = 2.2 µM; c(BC) = 2.5 µM. c) Fluorescence intensity at 427 nm (λ_{ex} = 378 nm) after the attainment of the equilibrium (300 s reaction time) as a function of 1-AdOH concentration at 25 °C; c(CB7) = 2.0 µM; c(MDAP) = 3.0 µM.

4.1. Direct binding assay (DBA)

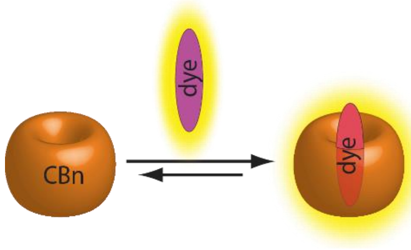

Scheme S 1. Schematic representation of a direct binding assay (DBA).

In a fluorescence-based direct binding assay (DBA), the host is titrated to a fluorescent guest and the reaction can be followed due to the change in the spectroscopic properties of the fluorescent guest triggered by the binding event. The intensity change can be fitted to determine the binding constant following Equation S1 - S5.

$$\mathrm{H + D \rightleftarrows HD} \qquad K_a^{HD} = \frac{[\mathrm{HD}]}{[\mathrm{H}][\mathrm{D}]} \qquad \text{Eq. S1+S2}$$

$$[\mathrm{H}]_0 = [\mathrm{HD}] + [\mathrm{H}] \qquad [\mathrm{D}]_0 = [\mathrm{HD}] + [\mathrm{D}] \qquad \text{Eq. S3+S4}$$

$$I_t = I^0 + I^{\mathrm{HD}} \cdot [\mathrm{HD}] + I^{\mathrm{D}} \cdot [\mathrm{D}] \qquad \text{Eq. S5}$$

4.2. Indicator displacement assay (IDA)

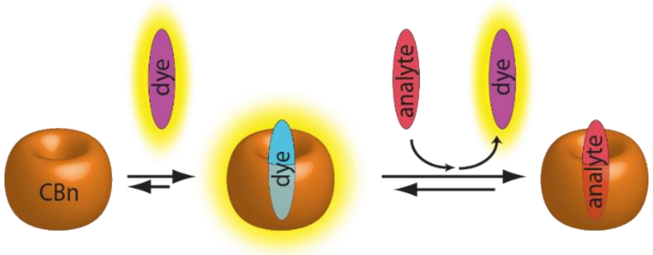

Scheme S 2. Schematic representation of an indicator displacement assay (IDA).

In an IDA, an indicator dye is equilibrated with the host to form a host•dye complex. Subsequently, a competitive guest is introduced into the system causing the displacement of the indicator from the host and the formation of a host•guest complex, which in turn modulates the optical signal. The assumption that only the formation of 1:1 complexes between indicator and host as well as competitor and host is made. The intensity change can be fitted to determine the binding constant (Equation S6 - S16).

$$\mathrm{HD + G \rightleftarrows HG + D} \qquad \text{Eq. S6}$$

$$\mathrm{H + D \rightleftarrows HD} \qquad \mathrm{H + G \rightleftarrows HG} \qquad \text{Eq. S7 + S8}$$

$$K_a^{HD} = \frac{[\mathrm{HD}]}{[\mathrm{H}][\mathrm{D}]} \qquad K_a^{HG} = \frac{[\mathrm{HG}]}{[\mathrm{H}][\mathrm{G}]} \qquad \text{Eq. S9 + S10}$$

$$[\mathrm{H}]_0 = [\mathrm{HD}] + [\mathrm{H}] + [\mathrm{HG}] \qquad \text{Eq. S11}$$

$$[\mathrm{D}]_0 = [\mathrm{HD}] + [\mathrm{D}] \qquad [\mathrm{G}]_0 = [\mathrm{HG}] + [\mathrm{G}] \qquad \text{Eq. S12 + S13}$$

$$[\mathrm{HD}] = \frac{K_a^{HD} \cdot [\mathrm{H}]}{1 + K_a^{HD} \cdot [\mathrm{H}]}[\mathrm{D}]_0 \qquad [\mathrm{HG}] = \frac{K_a^{HG} \cdot [\mathrm{H}]}{1 + K_a^{HG} \cdot [\mathrm{H}]}[\mathrm{G}]_0 \qquad \text{Eq. S14 + S15}$$

$$I_t = I^0 + I^{\mathrm{HD}} \cdot [\mathrm{HD}] + I^{\mathrm{D}} \cdot [\mathrm{D}] \qquad \text{Eq. S16}$$

[H] – host concentration at equilibrium, $[\mathrm{H}]_0$ – initial host concentration, [D] – dye concentration at equilibrium, $[\mathrm{D}]_0$ – initial dye concentration, [G] – guest concentration at equilibrium, $[\mathrm{G}]_0$ – initial guest concentration, [HD] – host·dye concentration at equilibrium, [HG] – host·guest concentration at equilibrium, K_a^{HD} – binding constant for the association of the HD complex, K_a^{HG} – binding constant for the association of the HG complex, I^0– background signal, I^{HD} – signal from HD complex, I^D – signal from free dye, I_t – observable signal as a function of time.

5. Packing Coefficients (PCs)

Table S 1. Guest volumes and packing coefficients (PCs) of the corresponding CB*n*•guest complexes. Hydroxygroups were calculated with a van der Waals (vdW) volume of 8 Å according to ref.[5]. The chemical structures of selected guests investigated for volume calculations are shown. The portions of the guest structures that are not located in the inner cavity of CB7 or CB8 are shown in red and with dashed lines. These moieties were removed before computing the vdW volume of the guests, and thus, do not affect the PC values.

		**CB7** inner cavity volume: 242 Å^3 [a] 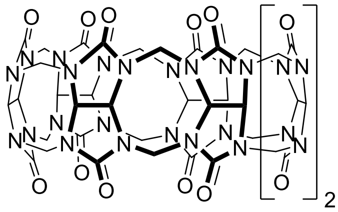		**CB8** inner cavity volume: 367 Å^3 [a] 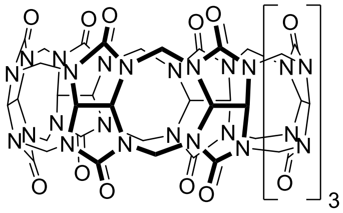	
molecule	vdW volume (Å^3)	volume buried in CB7 cavity (Å^3)	PC for CB7•guest complex	volume buried in CB8 cavity (Å^3)	PC for CB8•guest complex
**1-AdOH**	153	153 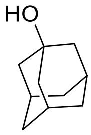	63%	153 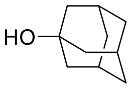	42%
**4-DAOH**	200	192 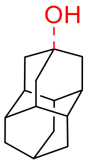	79%	200 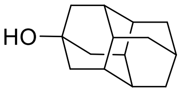	54%
**4,9-DA(OH)$_2$**	208	192 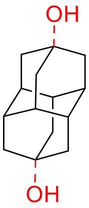	79%	208 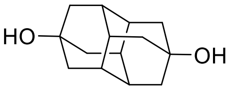	57%
**3,9-TA(OH)$_2$**	246	no inclusion complex formed with CB7		220 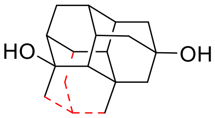	60%
**FeCp$_2$OH**	163	155 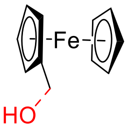	64%	155 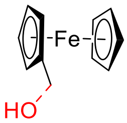	42%

[a] Inner cavity volume taken from ref.[6].

6. Comparison of the two applied multi-level approaches

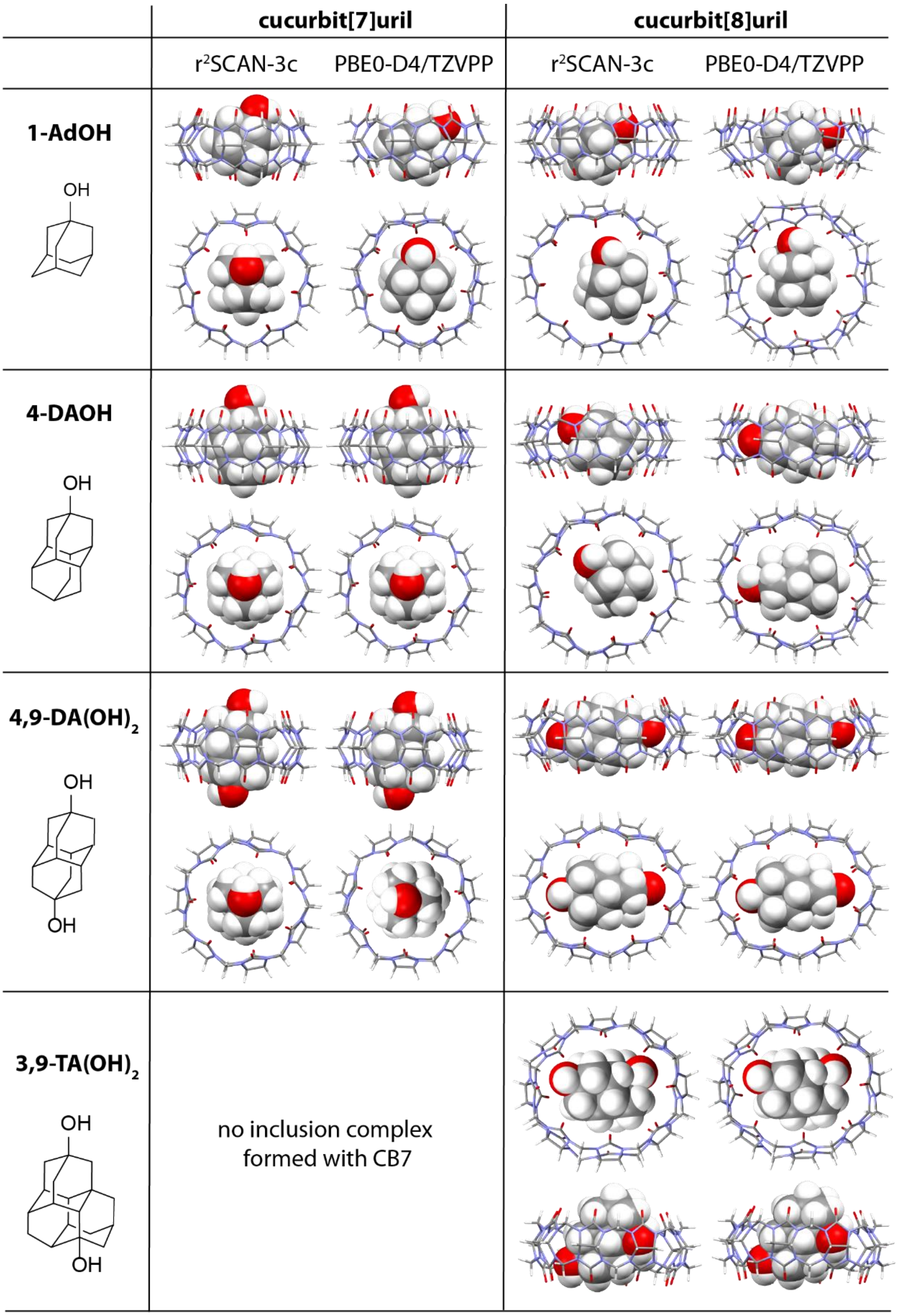

Figure S 11. Comparison of the quantum mechanically calculated structures for the within this work investigated complexes with the two different computational protocols, *i.e.*, r^{2}SCAN-3c and PBE0-D4/def2-TZVPP. The structures shown have the largest contribution to the calculated binding free energy.

7. Comparison of ΔG_{exp} and ΔG_{calc}

In order to be able to compare the experimentally and computationally determined energy values, all computationally obtained energy values were converted to standard conditions, namely 1 M in solution, as has been advocated by Ben-Naim previously.[7] For a more detailed explanation, we refer the reader to the work of Gilson, Grimme, and Nau[8] as well as to the COSMO*therm* reference manual.[9]

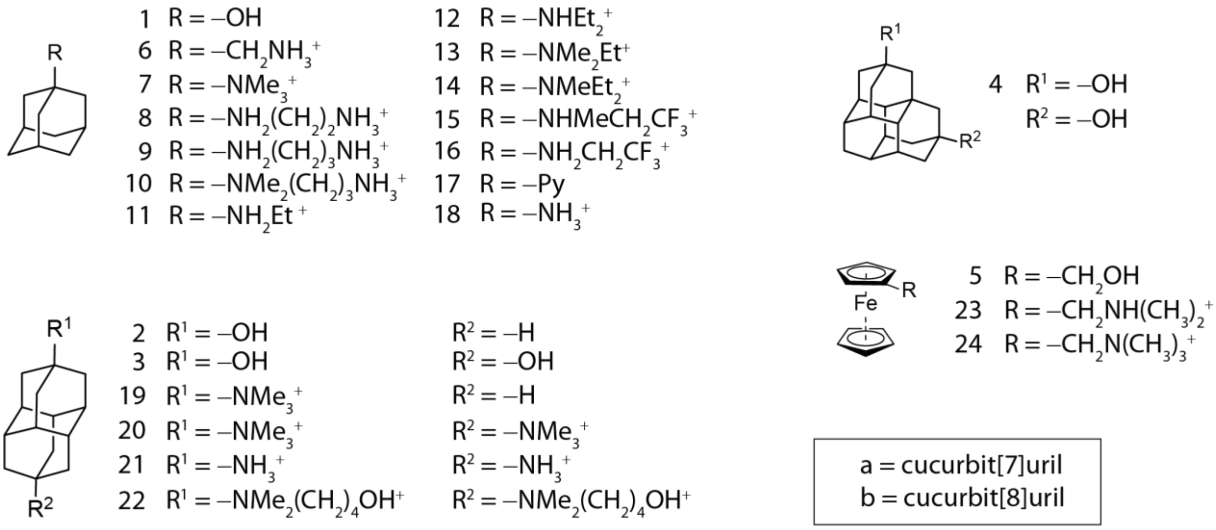

Figure S 12. Chemical structures of water-soluble guests listed in Table S 3 and S 4.

Table S2. Comparison of experimentally obtained values (ΔG_{exp}, ΔH_{exp}, and $-T\Delta S_{exp}$) in water at 25 °C and by the two applied multi-level approaches (r^2SCAN-3c and PBE0-D4/TZVPP) computationally obtained association free energies (ΔG_{calc}) of guest molecules in CB*n*. Further, single-point energies (ΔE), dispersion contribution (ΔE_{LD}), thermostatistical (ΔG_{mRRHO}) and solvation ($\Delta\delta G_{solv}$) contributions are listed. All values are given in kcal mol^{-1}.

host•guest complex	experiment			r^2SCAN-3c					PBE0-D4/TZVPP				
	ΔG_{exp}	ΔH_{exp}	$-T\Delta S_{exp}$	ΔG_{calc}	ΔE [a]	ΔE_{LD}	$\Delta\delta G_{solv}$ [b]	ΔG_{mRRHO} [c]	ΔG_{calc}	ΔE [d]	ΔE_{LD}	$\Delta\delta G_{solv}$ [b]	ΔG_{mRRHO} [e]
CB7•1-AdOH	–14.2	–19.0	4.8	–15.9	–30.5	–13.2	–0.3	14.9	–12.3	–29.8	–27.6	–0.2	17.7
CB7•4-DAOH	–9.3	–12.0	2.8	–9.9	–33.5	–15.4	6.4	17.3	–10.2	–26.1	–30.7	–3.5	19.4
CB7•4,9-DA(OH)$_2$	–9.6	–12.6	3.0	–8.3	–32.9	–15.7	7.4	17.3	–9.2	–26.3	–31.5	–2.5	19.6
CB7•FeCp$_2$OH	–12.8	–21.1	8.3	–12.6	–37.0	–14.4	7.3	17.1	–13.5	–33.7	–32.2	–0.9	21.1
CB8•1-AdOH	–9.3	–8.1	–1.2	–10.0	–25.1	–9.5	1.0	14.0	–4.3	–21.6	–18.8	2.2	15.1
CB8•4-DAOH	–9.1	–7.8	–1.2	–13.5	–35.1	–13.6	6.3	15.3	–12.4	–29.0	–27.3	1.0	15.5
CB8•4,9-DA(OH)$_2$	–9.9	–7.7	–2.3	–10.6	–41.4	–14.9	15.2	15.6	–10.1	–33.6	–30.7	6.3	17.2
CB8•3,9-TA(OH)$_2$	–9.5	–12.7	3.2	–9.1	–44.9	–15.8	19.1	16.6	–9.8	–35.5	–31.5	7.6	18.1
CB8•FeCp$_2$OH	–9.0	–13.1	4.2	–10.8	–29.5	–10.3	3.5	15.1	–10.0	–30.7	–25.9	3.6	17.0

[a] r^2SCAN-3c. $\Delta E = \Delta E_{el} + \Delta E_{LD}$. [b] COSMO-RS($H_2O$). [c] 298 K, GFN2-xTB. [d] PBE0-D4/def2-TZVPP. $\Delta E = \Delta E_{el} + \Delta E_{LD}$. [e] 298 K, PBEh-3c.

Table S 3. Comparison of experimentally obtained association free energy (ΔG_{exp}) values in water at 25 °C and by either DFT or M2 methods computationally obtained association free energies (ΔG_{calc}) of guest molecules in CB*n*, corresponding to Manuscript Figure 7a. a = CB7; b = CB8. Guest numbers are associated to the chemical structures shown in Figure S 12.

guest	host	this work			ref.[10]		ref.[11]		ref.[4a]		ref.[12]	
		ΔG_{calc} [a] (DFT)	ΔG_{calc} [b] (DFT)	ΔG_{exp} [c]	ΔG_{calc} [d] (DFT)	ΔG_{exp} [e]	ΔG_{calc} [f] (M2)	ΔG_{exp} [c]	ΔG_{calc} [g] (M2)	ΔG_{exp} [c]	ΔG_{calc} [g] (M2)	ΔG_{exp} [c]
1	a	–15.9	–12.3	–14.2			–14.3	–14.1				
1	b	–10.0	–4.3	–9.3								
2	a	–9.9	–10.2	–9.3								
2	b	–13.5	–12.4	–9.1								
3	a	–8.3	–9.2	–9.6								
3	b	–10.6	–10.1	–9.9								
4	b	–9.1	–9.8	–9.5								
5	a	–12.6	–13.5	–12.8			–9.5	–12.9	–7.7	–12.9	–10.5	–12.9
5	b	–10.8	–10.0	–9.0								
6	a						–19.3	–20.4[h]				
7	a				–30.0	–16.7						
8	a				–38.8	–18.2	–17.2	–21.5[h]				
9	a				–37.0	–17.9						
10	a				–36.8	–17.5						
18	a				–28.3	–17.2	–18.2	–19.4				
20	a				–41.2	–20.8						
21	b				–28.2	–16.2						
23	a						–12.5	–16.8			–14.6	–16.8
24	a						–12.2	–17.2	–14.6	–17.2	–14.5	–17.2

[a] r^{2}SCAN-3c. [b] PBE0-D4/TVZPP. [c] Determined by ITC in water at 25 °C. [d] DFT-D3 with BLYP-D3/def2-TZVPP. [e] Determined by NMR in 50 mM NaO_2CCD_3 buffer, pD 4.74, at 25 °C. [f] Calculations with finite-difference Poisson-Boltzmann (FDPB) grids, academic Lennard-Jones parameters, and dielectric cavity radii based on commercial Lennard-Jones σ values. [g] M2, local energy minima were identified with the Tork search algorithm, HA/MS method, CHARMM force field, Born model corrected with PB/surface area model, see also ref.[13]. [h] Due to slow equilibrium, ΔG_{exp} values were determined by NMR competition technique without error determination. All energy values were referenced to a standard concentration of 1 mol L^{-1}.

Table S 4. Comparison of experimentally obtained binding constants (K_a) for CB7 and CB8 complexes, corresponding to Manuscript Figure 7b.

guest	this work		ref.[14]		ref.[15]		ref.[16]	
	K_a(CB7•guest) (M^{-1}) [b]	K_a(CB8•guest) (M^{-1}) [b]	K_a(CB7•guest) (M^{-1}) [d]	K_a(CB8•guest) (M^{-1}) [d]	K_a(CB7•guest) (M^{-1}) [d]	K_a(CB8•guest) (M^{-1}) [d]	K_a(CB7•guest) (M^{-1}) [e]	K_a(CB8•guest) (M^{-1}) [e]
1	$2.6 \cdot 10^{10}$ [a]	$6.2 \cdot 10^{6}$						
2	$6.8 \cdot 10^{6}$	$4.4 \cdot 10^{6}$						
3	$1.1 \cdot 10^{7}$	$1.8 \cdot 10^{7}$						
5	$2.5 \cdot 10^{9}$ [c]	$3.6 \cdot 10^{6}$						
7							$1.7 \cdot 10^{12}$	$9.7 \cdot 10^{10}$
8			$2.4 \cdot 10^{13}$	$2.2 \cdot 10^{10}$				
10			$6.8 \cdot 10^{12}$	$1.7 \cdot 10^{12}$				
11			$8.7 \cdot 10^{11}$	$1.4 \cdot 10^{9}$				
12			$1.2 \cdot 10^{11}$	$3.1 \cdot 10^{9}$				
13			$7.0 \cdot 10^{11}$	$2.8 \cdot 10^{11}$				
14			$3.2 \cdot 10^{11}$	$1.1 \cdot 10^{11}$				
15			$1.1 \cdot 10^{11}$	$1.3 \cdot 10^{9}$				
16			$5.9 \cdot 10^{11}$	$1.0 \cdot 10^{9}$				
17			$2.0 \cdot 10^{12}$	$2.0 \cdot 10^{9}$			$2.0 \cdot 10^{12}$	$2.0 \cdot 10^{9}$
18							$4.2 \cdot 10^{12}$	$8.2 \cdot 10^{8}$
19			$8.0 \cdot 10^{11}$	$2.7 \cdot 10^{12}$				
20					$1.9 \cdot 10^{15}$	$2.0 \cdot 10^{12}$		
21					$1.3 \cdot 10^{11}$	$8.3 \cdot 10^{11}$		
22			$1.9 \cdot 10^{15}$	$1.3 \cdot 10^{13}$				

[a] Determined by fluorescence-based IDA titration in water at 25 °C. [b] Determined by ITC in water at 25 °C. [c] Determined by multistep ITC in water at 25 °C. [d] Determined by competitive NMR titrations in 50 mM NaO_2CCD_3 buffer, pD 4.74, at 25 °C. [e] Determined by competitive NMR experiments in deuterated water at 25 °C.

8. Literature data for CB7•guest complexes

Table S 5. Values collected during a literature survey on the entropic ($-T\Delta S_{exp}$) and enthalpic (ΔH_{exp}) contributions to the overall free association energy ΔG_{exp} for CB7 with high ($K_a > 10^9$ M^{-1}), medium (10^6 $M^{-1} < K_a < 10^9$ M^{-1}), and low ($K_a < 10^6$ M^{-1}) binders at 25±5 °C in solution with up to 10 mM salt content, corresponding to Manuscript Figure 6.

	guest	T (°C)	log K_a	ΔH_{exp} (kcal mol^{-1})	$-T\Delta S_{exp}$ (kcal mol^{-1})	ΔS_{exp} (cal mol^{-1} K^{-1})
CB7 high affinity guests	$FeCp_2OH$ [11]	25	9.5	−21.5	8.6	−28.9
	$FeCp_2OH$ [17]	25	9.5	−21.0	8.1	−27.2
	$FeCp_2OH$ [4a]	25	9.5	−21.5	6.2	−20.9
	$FeCp_2CH_2NMe_3^+$ [11]	25	12.6	−21.5	4.3	−14.4
	$FeCp_2CH_2NMe_3^+$ [4a]	25	12.6	−21.3	−4.1	13.6
	$FeCp_2CH_2NMe_3^+$ [17]	25	12.6	−21.0	−4.3	14.4
	$FeCp_2CH_2NHMe_2^+$ [11]	25	12.4	−21.0	4.1	−13.8
	$FeCp_2CH_2NHMe_2^+$ [17]	25	12.3	−21.3	−4.1	13.6
	1,1'-bis(trimethylamino)methyl-ferrocene [11]	25	15.5	−21.5	0.5	−1.7
	1,4-bis(hydroxylmethyl)-bicyclo[2.2.2]octane [11]	25	9.8	−15.8	2.4	−8.0
	1,4-bis(aminomethyl) bicyclo[2.2.2]octane^{2+} [11]	25	14.3	−15.6	−3.9	13.1
	1,4-dimethylpropane-1,3-diamino-bicyclo[2.2.2]octane [11]	25	15.1	−16.3	−4.3	14.4
	AdOH [11]	25	10.4	−19.0	4.9	−16.4
	$AdNH_3^+$ [11]	25	14.2	−19.3	−0.1	0.3
	$AdNH_2Me^+$ [11]	25	15.0	−21.9	1.7	−5.7
	1-(2-aminoethyl)adamantane^{2+} [11]	25	15.7	−20.1	−1.4	4.7
	2-$AdNH_3^+$ [11]	25	14.0	−19.5	0.4	−1.4
	N,N'-bis(aminoethyl)-1,6-hexane-diamine^{4+} [4a]	25	11.2	−8.8	−6.5	21.8
	1,6-hexanediamine^{2+} [4a]	25	9.3	−7.9	−4.9	16.3
	aminomethylcyclohexane$^+$ [4a]	25	11.1	−13.8	−1.3	4.5
	4-aminomethyl-Phe-Gly-Gly [18] [a]	27	9.0	−14.2	1.8	−6.0
CB7 medium affinity guests	nandrolone [19]	25	7.1	−12.5	2.9	−9.6
	nandrolone [19] [a]	25	6.6	−9.8	0.8	−2.7
	fenchol [20]	25	6.7	−10.1	1.0	−3.3
	spermine^{4+} [4a]	25	8.7	−6.5	−5.3	17.9
	1-methyl-3[[4-[(3-methylimidazol-3-ium-1-yl)methyl]phenyl]methyl] imidazol-1-ium [21]	25	6.4	−10.2	−4.8	16.0
	(+)-camphor [22]	25	7.3	−21.5	11.5	−38.7
	BC [23]	25	7.4	−9.1	−0.8	2.8
	6-methoxy-1-methylquinolinium [24]	25	6.3	−8.8	0.3	−0.9
	L-Phe [4a]	25	6.3	−10.4	1.8	−6.2
	hexadecyltrimethyl ammonium bromide [25]	25	6.4	−10.0	−1.5	4.9
	4-^{t}Bu-Phe [18] [a]	27	6.6	−14.5	5.4	−18.0
	4-aminomethyl-Phe [18] [a]	27	6.3	−4.2	−4.5	15.0

	Phe-Gly-Gly [18] [a]	27	6.5	−13.4	4.4	−14.7
	Phe-Gly-Gly [26] [b]	27	6.2	−10.8	2.3	−7.6
	Gly-Tyr-Gly [26]	27	6.4	−17.5	8.7	−28.9
	4-^{t}Bu-Phe-Gly-Gly [18] [a]	27	6.7	−16.2	7.1	−23.7
	Gly-4-aminomethyl Phe-Gly [18] [a]	27	6.3	−8.2	−0.5	1.7
	N-(furan-2-ylmethyl)prop-2-en-1-amine [27] [a]	25	6.5	−8.2	−0.6	2.0
	N-((5-methylfuran-2-yl)methyl)prop-2-en-1-amine [27] [a]	25	6.0	−5.9	−2.3	7.7
	6-chloro-1,2,3,6,7,7a-hexahydro-3a,6-epoxyisoindole [27] [a]	25	6.8	−8.8	−0.5	1.7
	1,2,3,6,7,7a-hexahydro-3a,6-epoxyisoindole [27] [a]	25	6.8	−7.1	−2.2	7.4
	N-((5-bromofuran-2-yl)methyl)prop-2-en-1-amine [27] [a]	25	6.9	−6.1	−2.1	7.1
	6-bromo-1,2,3,6,7,7a-hexahydro-3a,6-epoxyisoindole [27] [a]	25	6.5	−9.0	0.2	−0.6
	6-methyl-1,2,3,6,7,7a-hexahydro-3a,6-epoxyisoindole [27] [a]	25	6.8	−7.9	−1.3	4.3
	recombinant insulin serum [26]	27	6.2	−10.8	2.3	−7.6
	1-methyl-[4,4'-bipyridin]-1-ium [28] [a]	27	6.1	−5.5	−2.8	9.3
	4-aminomethyl-Phe-Met [29] [a]	27	8.7	−10.5	1.8	−6.0
	MV [28] [a]	27	6.8	−4.0	−5.3	17.7
	1-(3-ammoniopropyl)-1'-methyl-[4,4'-bi-pyridine]-1,1'-diium [28] [a]	27	7.2	−4.2	−5.6	18.6
	geranylamine [20]	25	6.5	−9.5	0.6	−2.0
	cadaverine [30] [b]	25	6.6	−4.1	−5.1	17.1
	tyramine [30] [b]	25	6.4	−8.5	−0.2	0.6
	dopamine [31]	25	5.7	-4.7	−3.0	10.2
CB7 low affinity guests	cyclopentanone [4a]	25	5.6	−9.6	1.9	−6.5
	eucalyptol [20]	25	5.9	−8.9	0.9	−2.9
	epinephrine[31]	25	4.2	−2.7	3.1	−10.3
	serotonin[31]	25	4.8	−3.7	−2.9	9.9
	$BaCl_2$[32]	25	4.8	−3.8	−2.7	9.0
	CsCl[32]	25	3.5	−2.3	−2.5	8.2
	RbCl[32]	25	3.4	−2.4	−2.3	7.7
	KCl[32]	25	3.3	−2.1	−2.4	8.1
	$CaCl_2$[32]	25	4.0	−2.3	−3.1	10.5
	acetone[33]	25	2.8	−3.1	−0.2	0.8
	pyrrole[33]	25	3.2	−7.2	2.6	−8.8
	cyclopentanone[33]	25	5.6	−9.8	2.2	−7.2
	DMF[33]	25	2.8	−5.3	1.4	−4.8
	DMSO[33]	25	2.1	−3.3	0.5	−1.6
	$SrCl_2$[32]	25	4.3	−3.4	−2.5	8.2
	Phe[18] [a]	27	5.1	−7.6	0.7	−2.3
	Gly-Phe-Gly[18] [a]	27	5.4	−9.8	2.4	−8.0
	L-Tyr	25	4.3	−5.2	−0.7	2.5
	N-((5-chlorofuran-2-yl)methyl)prop-2-en-1-amine	25	5.7	−6.7	−1.0	3.4
	L-Lys[34] [c]	25	2.3	−1.1	−2.1	7.1

L-Phe[34] [c]	25	5.3	−7.3	0.1	−0.5
L-Trp[34] [c]	25	3.1	−6.9	2.7	−9.1
L-Tyr[34] [c]	25	4.2	−6.6	0.9	−3.0
L-Trp[30] [b]	30	3.3	−6.0	1.5	−5.0
histamine[30] [b]	30	4.3	−2.3	−3.6	11.9
agmatine[30] [b]	30	5.9	−4.9	−3.3	10.9
L-Tyr[30] [b]	30	4.3	−6.5	0.6	−2.0
1,4-butandiamine[30] [b]	30	5.5	−3.3	−4.3	14.2
D/L-lysine[30] [b]	30	2.9	−4.1	1.2	−3.9
tryptamine[30] [b]	30	4.7	−8.3	1.8	−5.9
L-Arg[30] [b]	30	2.5	−1.2	−2.2	7.2
putrescine[30] [b]	30	5.5	−3.3	1.0	−3.4

[a] Data measured in 10 mM phosphate buffer, pH 7.0. [b] Data measured in 10 mM ammonium acetate buffer, pH 6.0. [c] Data measured in water at pH 6.0.

9. References

[1] M. T. H. Khan, *Bioactive Heterocycles IV, Vol. 10*, Springer-Verlag, Berlin Heidelberg, **2007**.

[2] S. Yi, A. E. Kaifer, *J. Org. Chem.* **2011**, *76*, 10275-10278.

[3] S. Sinn, E. Spuling, S. Bräse, F. Biedermann, *Chem. Sci.* **2019**, *10*, 6584-6593.

[4] a) M. V. Rekharsky, T. Mori, C. Yang, Y. H. Ko, N. Selvapalam, H. Kim, D. Sobransingh, A. E. Kaifer, S. Liu, L. Isaacs, W. Chen, S. Moghaddam, M. K. Gilson, K. Kim, Y. Inoue, *Proc. Natl. Acad. Sci.* **2007**, *104*, 20737; b) D. Shetty, J. K. Khedkar, K. M. Park, K. Kim, *Chem. Soc. Rev.* **2015**, *44*, 8747-8761.

[5] Y. H. Zhao, M. H. Abraham, A. M. Zissimos, *J. Org. Chem.* **2003**, *68*, 7368-7373.

[6] W. M. Nau, M. Florea, K. I. Assaf, *Isr. J. Chem.* **2011**, *51*, 559-577.

[7] a) A. Ben-Naim, *J. Phys. Chem.* **1978**, *82*, 792-803; b) A. Ben-Naim, *Solvation thermodynamics*, Plenum Press, New York, **1987**.

[8] K. I. Assaf, M. Florea, J. Antony, N. M. Henriksen, J. Yin, A. Hansen, Z.-w. Qu, R. Sure, D. Klapstein, M. K. Gilson, S. Grimme, W. M. Nau, *J. Phys. Chem. B* **2017**, *121*, 11144-11162.

[9] F. Eckert, *Version C3.0, Release17.01* **2015**, *17*, 1999-2016.

[10] J. Hostaš, D. Sigwalt, M. Šekutor, H. Ajani, M. Dubecký, J. Řezáč, P. Y. Zavalij, L. Cao, C. Wohlschlager, K. Mlinarić-Majerski, L. Isaacs, R. Glaser, P. Hobza, *Chem. Eur. J.* **2016**, *22*, 17226-17238.

[11] S. Moghaddam, C. Yang, M. Rekharsky, Y. H. Ko, K. Kim, Y. Inoue, M. K. Gilson, *J. Am. Chem. Soc.* **2011**, *133*, 3570-3581.

[12] S. Moghaddam, Y. Inoue, M. K. Gilson, *J. Am. Chem. Soc.* **2009**, *131*, 4012-4021.

[13] W. Chen, C.-e. Chang, M. K. Gilson, *J. Am. Chem. Soc.* **2006**, *128*, 4675-4684.

[14] D. Sigwalt, M. Šekutor, L. Cao, P. Y. Zavalij, J. Hostaš, H. Ajani, P. Hobza, K. Mlinarić-Majerski, R. Glaser, L. Isaacs, *J. Am. Chem. Soc.* **2017**, *139*, 3249-3258.

[15] L. Cao, M. Šekutor, P. Y. Zavalij, K. Mlinarić-Majerski, R. Glaser, L. Isaacs, *Angew. Chem. Int. Ed.* **2014**, *53*, 988-993.

[16] S. Liu, C. Ruspic, P. Mukhopadhyay, S. Chakrabarti, P. Y. Zavalij, L. Isaacs, *J. Am. Chem. Soc.* **2005**, *127*, 15959-15967.

[17] W. S. Jeon, K. Moon, S. H. Park, H. Chun, Y. H. Ko, J. Y. Lee, E. S. Lee, S. Samal, N. Selvapalam, M. V. Rekharsky, V. Sindelar, D. Sobransingh, Y. Inoue, A. E. Kaifer, K. Kim, *J. Am. Chem. Soc.* **2005**, *127*, 12984-12989.

[18] L. A. Logsdon, C. L. Schardon, V. Ramalingam, S. K. Kwee, A. R. Urbach, *J. Am. Chem. Soc.* **2011**, *133*, 17087-17092.

[19] A. I. Lazar, F. Biedermann, K. R. Mustafina, K. I. Assaf, A. Hennig, W. M. Nau, *J. Am. Chem. Soc.* **2016**, *138*, 13022-13029.

[20] M. A. Romero, N. Basílio, A. J. Moro, M. Domingues, J. A. González-Delgado, J. F. Arteaga, U. Pischel, *Chem. Eur. J.* **2017**, *23*, 13105-13111.

[21] D. Jiao, F. Biedermann, O. A. Scherman, *Org. Lett.* **2011**, *13*, 3044-3047.

[22] D.-S. Guo, V. D. Uzunova, K. I. Assaf, A. I. Lazar, Y. Liu, W. M. Nau, *Supramol. Chem.* **2016**, *28*, 384-395.

[23] Z. Miskolczy, L. Biczók, *J. Phys. Chem. B* **2014**, *118*, 2499-2505.

[24] Z. Miskolczy, J. G. Harangozo, L. Biczók, V. Wintgens, C. Lorthioir, C. Amiel, *Photochem. Photobiol. Sci.* **2014**, *13*, 499-508.

[25] M. Pessêgo, J. A. Moreira, L. Garcia-Rio, *Chem. Eur. J.* **2012**, *18*, 7931-7940.

[26] J. M. Chinai, A. B. Taylor, L. M. Ryno, N. D. Hargreaves, C. A. Morris, P. J. Hart, A. R. Urbach, *J. Am. Chem. Soc.* **2011**, *133*, 8810-8813.
[27] A. Palma, M. Artelsmair, G. Wu, X. Lu, S. J. Barrow, N. Uddin, E. Rosta, E. Masson, O. A. Scherman, *Angew. Chem. Int. Ed.* **2017**, *56*, 15688-15692.
[28] G. A. Vincil, A. R. Urbach, *Supramol. Chem.* **2008**, *20*, 681-687.
[29] L. A. Logsdon, A. R. Urbach, *J. Am. Chem. Soc.* **2013**, *135*, 11414-11416.
[30] D. M. Bailey, A. Hennig, V. D. Uzunova, W. M. Nau, *Chem. Eur. J.* **2008**, *14*, 6069-6077.
[31] S. Kasera, L. O. Herrmann, J. d. Barrio, J. J. Baumberg, O. A. Scherman, *Sci. Rep.* **2014**, *4*, 6785.
[32] S. Zhang, L. Grimm, Z. Miskolczy, L. Biczók, F. Biedermann, W. M. Nau, *Chem. Commun.* **2019**, *55*, 14131-14134.
[33] F. Biedermann, V. D. Uzunova, O. A. Scherman, W. M. Nau, A. De Simone, *J. Am. Chem. Soc.* **2012**, *134*, 15318-15323.
[34] J. W. Lee, H. H. L. Lee, Y. H. Ko, K. Kim, H. I. Kim, *J. Phys. Chem. B* **2015**, *119*, 4628-4636.
